# Suppressed Protein Translation Caused by MSP‐8 Deficiency Determines Fungal Multidrug Resistance with Fitness Cost

**DOI:** 10.1002/advs.202412514

**Published:** 2024-12-16

**Authors:** Mi Zhou, Pengju Yu, Chengcheng Hu, Wenxia Fang, Cheng Jin, Shaojie Li, Xianyun Sun

**Affiliations:** ^1^ State Key Laboratory of Mycology Institute of Microbiology Chinese Academy of Sciences Beijing 100101 China; ^2^ National Institute for Radiological Protection China CDC Beijing 100088 China; ^3^ University of Chinese Academy of Sciences Beijing 100049 China; ^4^ Institute of Biological Sciences and Technology Guangxi Academy of Sciences Nanning Guangxi 530007 China

**Keywords:** antifungal drug, *Aspergillus fumigatus*, *Fusarium verticilioides*, helicase, multidrug resistance, *Neurospora crassa*, protein translation

## Abstract

Antifungal resistance, particularly the rise of multidrug‐resistance strains, poses a significant public health threat. In this study, the study identifies a novel multidrug‐resistance gene, *msp‐8*, encoding a helicase, through experimental evolution with *Neurospora crassa* as a model. Deletion of *msp‐8* conferred multidrug resistance in *N. crassa*, *Aspergillus fumigatus*, and *Fusarium verticillioides*. However, the transcript levels of genes encoding known drug targets or efflux pumps remain unaltered with *msp‐8* deletion. Interestingly, MSP‐8 interacted with ribosomal proteins, and this mutant displays compromised ribosomal function, causing translational disturbance. Notably, inhibition of protein translation enhances resistance to azoles, amphotericin B, and polyoxin B. Furthermore, MSP‐8 deficiency or inhibition of translation reduces intracellular ketoconazole accumulation and membrane‐bound amphotericin B content, directly causing antifungal resistance. Additionaly, MSP‐8 deficiency induces cell wall remodeling, and decreases intracellular ROS levels, further contributing to resistance. The findings reveal a novel multidrug resistance mechanism independent of changes in drug target or efflux pump, while MSP‐8 deficiency suppresses protein translation, thereby facilitating the development of resistance with fitness cost. This study provides the first evidence that MSP‐8 participates in protein translation and that translation suppression can cause multidrug resistance in fungi, offering new insights into resistance mechanisms in clinical and environmental fungal strains.

## Graphical abstract

This study identifies *msp‐8* as a novel multidrug‐resistance gene encoding a helicase in fungi, and reveals a previously unrecognized resistance mechanism. Dysfunction of MSP‐8 disrupts translation homeostasis, leading to reductions in intracellular ketoconazolelevels, membrane‐bound amphotericin B contents, and reactive oxygen species. Additionally, *msp‐8* dysfunction induces cell wall remodeling, collectively contributing to enhanced fungal multidrug resistance.

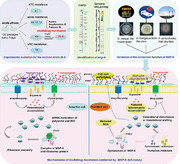

## 1 Introduction

Fungal infections represent a significant and often underestimated threat, with more than a billion people infected worldwide and nearly 3.75 million mortalities per year.^[^ ^1^ ^]^ Additionally, infections by fungal pathogens also pose a serious threat to crop production^[^ ^2^ ^]^ and biodiversity,^[^ ^3^ ^]^ garnering increasing attention. However, the available antifungal drugs used for the prevention and control of fungal disease are limited. According to their mode of action, the azoles, including ketoconazole (KTC), and the polyenes, such as amphotericin B (AmB), exert their antifungal effects by targeting the cell membrane. The echinocandins, such as caspofungin specifically interfere with the synthesis of β‐glucans in the cell wall, The peptidylpyrimidines, exemplified by polyoxin B (PoxB), act on the chitin of the cell wall. The frequent and prophylactic use of antifungal agents has led to the development of robust resistance in both medically and agriculturally important fungi.^[^ ^4^ ^]^ In recent years, there have been increasing reports of fungi developing multidrug resistance,^[^ ^5^, ^6^ ^]^ especially the emerging *Candida auris* and other *Candida* species with unknown resistance mechanisms exacerbating the threat of fungal infection.^[^ ^7^ ^]^ The limited drug targets and the rapid emergence of drug‐resistant strains present significant challenges for antifungal therapy.

Previous studies have shown that antifungal resistance often results from alterations in genes encoding drug targets or drug efflux pumps. In *Candida* species (eg *C. albicans*, *C. auriculata*, *C. glabra*), resistance to the azoles and echinocandins is often caused by upregulation of drug target genes, such as *FKS1* (the target gene of echinocandins) and *ERG11* (the target gene of azoles).^[^ ^8^ ^]^ This upregulation decreases drug sensitivity by increasing the target's abundance, thus reducing the effective concentration of the drug. In addition, intracellular drug concentrations can be lowered by upregulating efflux pump gene expression.^[^ ^8^ ^]^ In *Aspergillus fumigatus*, azole resistance is primarily associated with Cyp51A (Erg11 homologous protein) mutations, which reduce binding affinity of azoles to the Cyp51A protein.^[^ ^9^ ^]^ Transcriptional regulation of the drug efflux pump and target genes by transcription factors plays a vital role in response to antifungals. Gain‐of‐function mutations in transcription factors such as Upc2p or Tac1p can cause constitutively high expression of *ERG11* and drug pump coding genes *CDR1* and *CDR2*, respectively, contributing to drug resistance.^[^ ^8^ ^]^ Clinically, fungal pathogens often develop multidrug resistance by accumulating mutations across various drug targets or drug pump genes after period treatment by the azole and echinocandins.^[^ ^10^, ^11^ ^]^ Thus, the drug target and efflux pumps along with their regulatory networks, are widely recognized as key determinants of drug resistance. However, there are a growing number of clinical cases, including *A. fumigatus* isolates, that exhibit antifungal resistance without either cyp51A change or efflux pump gene upregulation, suggesting alternative or unknown resistance mechanisms to be further investigated.^[^ ^12^, ^13^ ^]^

With the emergence of multidrug resistance fungi like *Candida auris* (“super fungi”), which are resistant to multiple categories of antifungal drugs, understanding alternative resistance mechanisms has become imperative. Great efforts have been made to explore the mechanisms of multidrug resistance/tolerance. One mechanism by which this occurs is via the variation in drug targets or drug pump genes. Other mechanisms include the formation of biofilm and persister cells. In the model of biofilm resistance, antibiotic treatment eliminates the bulk of both biofilm and planktonic cells, leaving intact persisters. When antibiotic concentration drops, persisters repopulate the biofilm, which sheds off new planktonic cells, producing the relapsing biofilm infection.^[^ ^14^ ^]^ However, how the persisters form and how they obtain resistance to “everything” remains to be further studied. Besides, the emergence of hypermutator lineages can also cause multidrug resistance. It was reported in *C. glabrata* that one‐third of the 1300 isolates were identified as non‐susceptible to both an echinocandin and an azole, with approximately 55% of resistant clinical isolates having loss‐of‐function mutations in the DNA repair gene *MSH2*, accelerating the emergence of multidrug resistance.^[^ ^4^, ^15^ ^]^ However, hypermutation does not always correlate with multidrug resistance,^[^ ^16^ ^]^ as shown in other *C. glabrata* studies where 44.4% of *MSH2* gene mutations were not correlated with in vitro or in vivo antifungal resistance.^[^ ^16^ ^]^

A recent study highlights the role of translational mechanisms in antifungal resistance, with findings that only very few genes are controlled both by translational regulation and transcriptional regulation under fluconazole treatment, highlighting the importance of translation in response to drug stress.^[^ ^17^ ^]^ Protein synthesis with minimum errors and energy expenditure is required for the ribosomes with complete and appropriate subunits coordinated in specific ways.^[^ ^18^ ^]^ It is reported that most ribosomal proteins are synthesized from duplicated genes that generate different ribosome productions in response to changing growth conditions.^[^ ^18^ ^]^ Thus, ribosome heterogeneity and protein translation are also involved in environmental stress, including drug stress.^[^ ^18^, ^19^ ^]^ As ribosome profiling techniques have been used only since 2009, there are fewer studies on the translational level compared to the gene level, such as gene variation and transcription.^[^ ^17^, ^20^ ^]^ However, it is an indispensable and vital process in the mechanism of antifungal resistance.

Here, we employed *Neurospora crassa cdr4* knockout strain (*cdr4* ^KO^) whose major azole efflux pump is deleted, for laboratory evolution under azole stress. An evolved strain 2k‐6 was identified with an *msp‐8* mutation that was associated with multidrug resistance to KTC, AmB, and PoxB. Functional analysis confirmed that *msp‐8* deletion conferred multidrug resistance in pathogens such as *A. fumigatus* and *Fusarium verticillioides*. The protein MSP‐8, a predicted DEAD/DEAH‐box helicase, containing the Asp‐Glu‐Ala‐Asp (DEAD) motif, belongs to the superfamily 2 helicases involved in diverse RNA metabolism.^[^ ^21^, ^22^ ^]^ In this study, MSP‐8 interacted with ribosomal protein CYH‐2, and the deletion of *msp‐8* in *N. crassa* led to multidrug resistance, similar to the translation inhibitor cycloheximide (CHX) or anisomycin (ASM) treatment on the wild‐type (WT). Interestingly, MSP‐8 deficiency reduced intracellular KTC accumulation and membrane‐bound AmB content suggesting that translation arrest affected drug accumulation. In the *msp‐8* ^KO^ mutant, the transcript levels of purine‐cytosine permease PUP‐6 and several peptide transporters were decreased, implying reduced drug uptake due to *msp‐8* deletion. Additionally, remodeling cell wall with decreased the contents of chitin and β‐glucans, and reduced reactive oxygen species (ROS) generation indicated altered cellular components and physiological status contributing to multidrug resistance. Altogether, this study demonstrates that MSP‐8 deficiency causes multidrug resistance through translational regulation, presenting a novel resistance mechanism centered on translation.

## 2 Results

### 2.1 The *N. Crassa* Evolved Strain 2k‐6 Acquires Multidrug Resistance to the Azole, Amphotericin B (AmB), and Polyoxin B (PoxB)

**Figure 1:**
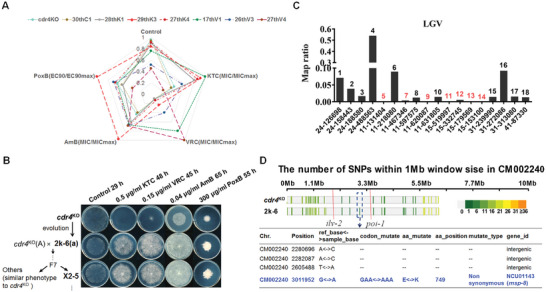
The multidrug resistance phenotype of 2k‐6 is associated with the MSP‐8 point mutation (E749K). A) Spider web diagram depicting changes in antifungal susceptibility profiles of the indicated strains. B) Schematic illustrating the inheritance stability of multidrug resistance in strain 2k‐6 via backcrossing. The multidrug resistance phenotype is determined by drug susceptibility testing at designated concentrations of different kinds of antifungals. Plates are incubated at 28 °C for the indicated time points. The antifungal drugs used in this study are as follows: KTC (ketoconazole), VRC (voriconazole), PoxB (polyoxin B), AmB (amphotericin B). C) CAPS assay performed with 18 markers as described by Randy Lambreghts et al.(2009) spaced across LGV. CAPS makers are named by their physical location given as <contig>‐<distance from start of contig in kb>) on the X‐axis. Map ratios estimated from band intensities for SNPs are shown on the Y‐axis. Map ratios below 0.01 are indicated red numbers. The mapping methodology is detailed in the Experimental Section. D) SNP information for the mapped mutation between *ilv‐2* (1828369) and *poi‐1* (3397430) on LGV, highlighted by a red line. The colors represent the frequency of SNPs. Source data are provided in File S1 (Supporting Information). The specific SNPs (dashed box) in the 2k‐6 strain are shown in the chart below.

Through experimental evolution^[^ ^23^ ^]^ of a *cdr4* ^KO^ strain—a mutant lacking the azole efflux pump gene *cdr4*—under azole exposure, six *cdr4* ^KO^ evolved populations (28thK1, 29thK3, 27thK4, 17thV1, 25thV3 and 27thV4) were obtained under KTC or voriconazole (VRC) stress. These populations were evaluated for resistance against standard antifungal drugs, including the azoles (KTC and VRC), AmB (a polyene), and PoxB (a chitin synthesis inhibitor), using a spot assay on solid media. As shown in Figure S1 (Supporting Information), the KTC‐evolved populations (28thK1, 29thK3, and 27thK4) displayed significant resistance to KTC, while VRC‐evolved populations (17thV1, 25thV3, and 27thV4) enhanced resistance to VRC. Notably, the 29thK3 population displayed increased resistance to AmB, with an inhibition rate of 39.90%, significantly lower than the 69.03% in the parent *cdr4* ^KO^ (Figure S1, Supporting Information). For PoxB, 29thK3, 27thK4, and 26thV3 showed significantly increased resistance, particularly 29thK3, with an inhibitory rate of 64.15%, distinctively lower than the 85.84% in *cdr4* ^KO^ (Figure S1, Supporting Information). A radar map was drawn by calculating MIC, EC_90_, and growth rate to visualize the adaptive levels to the four tested antifungal drugs and no drug. As shown in **Figure** **1**A, the population 29thK3 displayed appropriate fitness under drug‐free conditions (the control condition) and showed remarkable multidrug resistance, not only to the azoles but also to other categories of antifungal drugs including PoxB and AmB.

To elucidate the mechanisms underlying this multidrug resistance, we isolated a monokaryon spore 2k‐6 from the 29thK3 population. As shown in Figure 1B, strain 2k‐6 exhibited remarkably increased resistance to KTC, VRC, AmB, and PoxB, similar to the phenotype of 29thK3. Genetic stability tests confirmed the multidrug resistance in strain 2k‐6 through seven successive backcrosses with the *cdr4* ^KO^ parental strain (FGSC#11239, *mat* A), with resistance maintained across progeny (Figure 1B), indicating that the phenotype of multidrug resistance was genetically stable. Notably, all PoxB‐resistance progeny were bound up with resistant phenotype to both the azoles and AmB, suggesting that the resistance was due to either a single mutation site or several mutations in close genetic linkage.

To examine the contribution of the azole target ERG11 to the azole resistance in the strain 2k‐6, we determined nucleotide sequences, transcript levels, protein expression of *erg11*, and metabolite ergosterol accumulations in *cdr4* ^KO^ and 2k‐6 strains, revealing no significant differences (Figure S2A–C, Supporting Information). HPLC analysis indicated that intracellular KTC accumulation in 2k‐6 was significantly reduced, reaching only 30% of that in *cdr4* ^KO^ (Figure S2D, Supporting Information). Considering the major azole efflux pump gene *cdr4* is knocked out in stain 2k‐6, we investigated the transcriptional levels of *atrf* and *atrf‐2*, homologous genes of *cdr4*. Both genes were upregulated in 2k‐6 with or without KTC treatment (Figure S2E, Supporting Information). However, individual knockouts of these genes in 2k‐6 (2k‐6::*atrf* ^KO^ and 2k‐6::*atrf‐2* ^KO^) did not alter susceptibility to azole, AmB, or PoxB, indicating that PDR subfamily efflux pumps likely do not contribute to resistance in strain 2k‐6 (Figure S2F, Supporting Information). Thus, we hypothesized that reduced drug uptake may account for the diminished intracellular KTC levels observed. To evaluate the uptake and efflux capacities of strain 2k‐6, intracellular accumulations of rhodamine 6G (R6G), a mimicking substrate of ATP‐binding cassette (ABC) transporters, were measured by fluorescence‐activated cell sorting (FACS). As shown in Figure S2G (Supporting Information), the presence of glucose led to a marked reduction in fluorescence intensity for the WT strain by approximately 50%, serving as a control. In contrast, the fluorescence intensities of the 2k‐6 and *cdr4* ^KO^ strains remained relatively stable, indicating that the substrate efflux ability of strain 2k‐6 was similar to that of *cdr4* ^KO^ and significantly lower than that of WT. While prior to glucose addition, the fluorescence intensity of *cdr4* ^KO^ and 2k‐6 was 3.4 and 2.9 times higher than that of WT, respectively, and the 2k‐6 strain displayed a weaker fluorescence intensity compared to *cdr4* ^KO^, reaching 86.36% of that in *cdr4* ^KO^, indicating the 2k‐6 strain exhibits a relatively weaker drug uptake capacity compared to the *cdr4* ^KO^ strain.

### 2.2 The E749K Mutation in MSP‐8 is Associated with the Multidrug Resistance Phenotype of Strain 2k‐6

To identify the mutation responsible for the multidrug resistance phenotype in 2k‐6, we conducted a genetic cross with a Mauriceville strain (FGSC#2225) and examined the drug susceptibility of the progeny. They were categorized into two groups based on their phenotype: those exhibiting multidrug resistance to azole, AmB, and PoxB, and those showing sensitivity to the drug. We then performed cleaved amplified polymorphic sequence (CAPS) analysis with established genomic markers.^[^ ^24^ ^]^ By measuring the mapping ratio of linkage to the target SNP, we mapped it to a 1.57 Mbp region between the genetic location of markers *ilv‐2* (position 1828369) and *poi‐1* (position 3397430) of linkage group V (LGV) (Figure 1C; Figure S3A, Supporting Information). To identify the mutation site precisely, whole‐genome resequencing of 2k‐6 was performed. Comparison of the genome sequences between the ancestral *cdr4* ^KO^ strain and 2k‐6 within the genetically mapped region revealed three SNPs in the intergenic region, and a single nucleotide change within the open reading frame of *msp‐8* in LGV (Figure 1D; File S1, Supporting Information). A nonsynonymous mutation, GAA to AAA, at position 3011952 of LGV resulted in a single substitution of glutamate to lysine at position 749 (E749K) of MSP‐8 protein (Figure 1D; Figure S3B, Supporting Information). To confirm the association between the E749K mutation and the resistant phenotype, we analyzed the selected progeny of the cross between strain 2k‐6 and FGSC#2225. The resistant progeny (p4, p10, p11, p15, and p31) possessed the G‐to‐A mutation, while sensitive progeny (p2, p6, p9, and p20) lacked this mutation (Figure S3C, Supporting Information), confirming the correlation between the GAA‐to‐AAA point mutation in *msp‐8* and resistant phenotype.

Sequence alignments and phylogenetic analysis showed that MSP‐8 is highly conserved across eukaryotes among different fungal pathogens, with DEXDc and HELICc domains (Figure S3B,D, and File S2, Supporting Information). The E749K substitution of MSP‐8 occurs within the conserved motif II in the DEXDc domain, which is the core functional region for NTP/ATP binding and hydrolysis.^[^ ^22^, ^25^ ^]^

**Figure 2:**
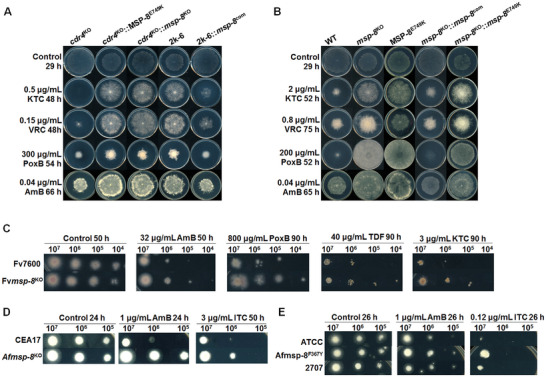
Identification of E749K mutation in MSP‐8 associated with multidrug resistance phenotype in the strain 2k‐6. A) Drug susceptibility test of the indicated *N. crassa* strains: the *msp‐8* knockout mutant (*cdr4* ^KO^::*msp‐8* ^KO^), the point mutant (*cdr4* ^KO^::MSP‐8^E749K^), the background strain *cdr4* ^KO^ mutant, the evolved mutant 2k‐6 and the *msp‐8* complementary strain in 2k‐6 (2k‐6::*msp‐8* ^com^). B) Drug susceptibility test of the *msp‐8* knockout mutant (*msp‐8* ^KO^), the point mutant (MSP‐8^E749K^), the *msp‐8* complementary strain (*msp‐8* ^KO^::*msp‐8* ^com^), and the point mutated *msp‐8* complementary strain (*msp‐8* ^KO^::*msp‐8* ^E749K^), against designated concentrations of different antifungals. Two microliter aliquots of conidial suspension (2 × 10^6^ conidia/mL) were inoculated in the center of plates (ϕ90‐mm) with or without the antifungals. The plates were then incubated at 28 °C for the indicated time. C) Drug susceptibility test of the *msp‐8* homologous gene knockout mutant (Fv*msp‐8* ^KO^) in *F. verticillioides* and the wild‐type strain (Fv7600). Two microliter aliquots of conidial suspension with gradient dilution (10^7^, 10^6^, 10^5^, 10^4^ conidia /mL) were inoculated onto PDA plates (ϕ150‐mm) with or without the antifungals. The plates were then incubated at 28 °C for the indicated time. D) Drug susceptibility test of the *msp‐8* homologous gene knockout mutant (Af*msp‐8* ^KO^) in *A. fumigatus* and the wild‐type strain (CEA17). E) Drug susceptibility test of the clinical isolated strain 2707 (containing point mutation of F367Y in Af*msp‐8*), the point mutant (Afmsp‐8^F367Y^), and the reference strain (ATCC), against designated concentrations of different antifungals. Two microliter aliquots of conidial suspension with gradient dilution (10^7^, 10^6^, 10^5^ conidia/mL) were inoculated in CM plates (ϕ90‐mm) with or without the antifungals. The plates were then incubated at 37 °C for the indicated time. All experiments were independently repeated at least three times. Abbreviations: TDF (Triadimefon), ITC (Itraconazole).

To verify the role of *msp‐8* and the E749K mutation in multidrug resistance of strain 2k‐6, the *msp‐8* knockout mutant and the point mutation of MSP‐8^E749K^ in *cdr4* ^KO^, along with the complementary strain of the 2k‐6 were constructed, respectively. As shown in **Figure** **2**A, both the *msp‐8* knockout mutant (*cdr4* ^KO^::*msp‐8* ^KO^) and the E749K mutation in *cdr4* ^KO^ (*cdr4* ^KO^::MSP‐8^E749K^) displayed the weaker growth than *cdr4* ^KO^ under normal culture condition, and significantly increased resistance to azoles, AmB and PoxB, similar to the phenotype of the strain 2k‐6. Quantitatively, compared to the growth rate of WT with 0.448 cm/h, 2k‐6 showed a slower rate of 0.347 cm/h similar to that of 0.365 cm/h in the *msp‐8* ^KO^ mutant (Figure S3E, Supporting Information). Thus, 2k‐6 and *msp‐8* ^KO^ exhibited a similar degree of fitness cost on standard culture media. Moreover, complementation of strain 2k‐6 with wild‐type *msp‐8* (2k‐6::*msp‐8* ^com^) restored drug sensitivity levels close to those of *cdr4* ^KO^. These results illustrated MSP‐8^E749K^ played a central role in multidrug resistance and associated fitness cost in strain 2k‐6. Further experiments in the WT strain involving *msp‐8* knockout (*msp‐8* ^KO^), E749K mutation (MSP‐8^E749K^), and complementation (*msp‐8* ^KO^::*msp‐8* ^com^) confirmed the role of MSP‐8 in conferring multidrug resistance to azoles, AmB, and PoxB at the expense of growth fitness in *N. crassa* (Figure 2B).

To explore the functional conservation of MSP‐8 homologs in other fungi, we constructed Fv*msp‐8* (FVEG_08023) and Af*msp‐8* (AFUA_2G16140) deleted mutants in the wild‐types of *F. verticillioides* (Fv4200) and *A. fumigatus* (CEA17), respectively. As shown in Figure 2C,D, the Fv*msp‐8* knockout mutant (Fv*msp‐8* ^KO^) increased resistance to AmB, PoxB, triadimefon (TDF), and KTC, while the Af*msp‐8* ^KO^ mutant elevated resistance to AmB and itraconazole (ITC), indicating functional conservation of MSP‐8 homologs in fungal drug resistance. Additionally, we isolated an *A. fumigatus* 2707 clinical strain with an F367Y mutation in MSP‐8 and constructed the point mutation Af*msp‐8* ^F367Y^. Both mutants showed increased resistance to ITC and AmB (Figure 2E), suggesting that *msp‐8* mutations may serve as drug resistance markers in pathogenic fungi.

### 2.3 Suppressed Protein Translation Caused by MSP‐8 Deletion Contributes to Multidrug Resistance

**Figure 3:**
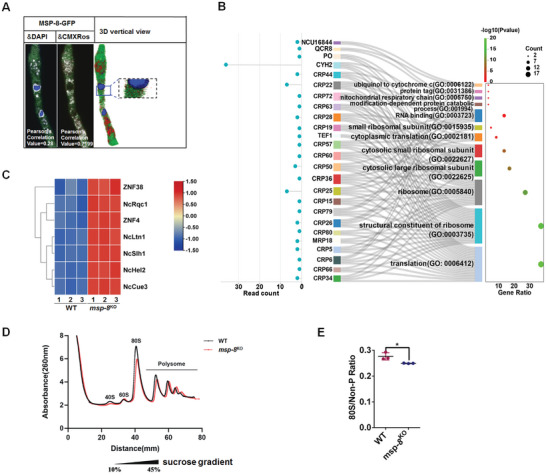
Deletion of MSP‐8 disrupts protein translation. A) Laser confocal microscopic images showing the distribution of MSP‐8‐GFP in *N. crassa* cells under control condition. DAPI staining is used to visualize the nucleus (blue). The merged 3D image of GFP and DAPI staining demonstrates non‐nuclear localization of MSP‐8‐GFP (Pearson's Correlation Value for co‐localization = 0.2813). Mitochondria are visualized using Red‐MitoTracker dye. B) Gene ontology (GO) analysis of the MSP‐8‐interacting proteins identified by Y2H, with the Sankey diagram on the left illustrating the connections between the interacting proteins and the enriched GO items. Source data are provided in File S3 (Supporting Information). C) Heat map comparison of the proteomic data showing the expression levels of RQC system proteins in WT and *msp‐8* ^KO^ strains. Colors represent the log_2_‐transformed fold changes in protein levels detected in the indicated strains. D,E) Representative polysome profiles (D) from WT and *msp‐8* ^KO^ strains, and quantification of the 80S monosome to non‐polysome (80S/Non‐P) ratios (E). Data are presented as mean ± SD. Statistical significance between the WT strain and *msp‐8* ^KO^ mutant is assessed using a two‐tailed *t*‐test. Values with 0.01 < *p* < 0.05 are marked with*. *p* = 0.0296, n = 3.

The biological function of MSP‐8 and its homologs remains uncharacterized. To investigate MSP‐8 function, we labeled MSP‐8 at the C‐terminus with GFP and found that MSP‐8 localized predominantly in the cytoplasm rather than in the nucleus (Pearson's Correlation Value = 0.2813) (**Figure** **3**A; Figure S4A, Supporting Information), and tended to colocalize with a mitotracker (Pearson's Correlation Value = 0.7199) (Figure 3A). Moreover, the green fluorescence did not change localization upon antifungal drug stress (Figure S4A, Supporting Information). These data indicated that MSP‐8 functioned mainly in the cytoplasm and mitochondria. A high‐throughput yeast two‐hybrid (Y2H) screening identified 58 MSP‐8 interacting proteins, over one‐third of which (22/58) are constituents of the ribosome and enriched in the “translation” category (GO: 0006412). Two mitochondrial electron transport‐related proteins, QCR8 and NCU16844, were also identified and enriched. Notably, ribosomal protein CYH‐2 was mapped with 36 read counts, the highest sequencing counts among the mapped proteins (Figure 3B; File S3, Supporting Information). Moreover, compared to the *msp‐8* ^KO^ and WT strains, the deletion of *cyh‐2* (*cyh‐2* ^KO^) displayed the weakest growth (Figure S3E, Supporting Information) since CYH‐2 is a conserved and important ribosomal protein, while MSP‐8 may only impair some of the ribosome function, and it increased resistance to KTC and AmB (Figure S4B, Supporting Information), suggesting CYH‐2 dysfunction caused by the deletion of *msp‐8* contributed to antifungal resistance. To validate the interaction of MSP‐8 with ribosomal proteins, we tagged CYH‐2 with FLAG at its N‐termini and co‐expressed it in the MSP‐8‐5×MYC expressed strain. Using Co‐IP assays, we confirmed the interaction between MSP‐8 and CYH‐2 (Figure S4C, Supporting Information). CYH‐2 is the binding site of cycloheximide (CHX), an inhibitor to the translocation step of translation, causing stalling.^[^ ^26^ ^]^ Thus, MSP‐8 interacted with the ribosomal protein indicating MSP‐8 deficiency may disrupt the protein translation process.

Using Tandem Mass Tags (TMT) proteome sequencing, we analyzed differentially expressed proteins (Fold change>1.3 or <0.769, P<0.05) between WT and *msp‐8* ^KO^. Gene ontology (GO) analysis of the differently expressed proteins showed that the enriched items included ribosomal quality control (RQC) complexes, ATP binding cassette (ABC) transporter activity, and extracellular regions (Figure S4D, Supporting Information). Notably, the RQC complex components NcRqc1 (Q7RX23), NcLtn1 (Q7S834), and ZNF‐28 (Q7S469), linked to rescuing stalled ribosome (GO: 0072344) and translation regulation (GO: 0006417) were regulated (Figure S4D,E, Supporting Information). They were displayed in an interaction network with four other proteins, including ZNF‐4 (Q7SER9) involved in peptidyl‐tRNA hydrolase that releases stalled peptides from ribosomes, and the three subunits of ribosome‐associated quality control trigger (RQT) complex ZNF‐38 (Q7S389), NcSlh1 (Q7S8K0) and NcCue3 (Q7S3G0) (Figure S4E, File S4, Supporting Information). In eukaryotes, the RQC system represents a rescue pathway triggered upon translational disturbance or translational stalling.^[^ ^27^, ^28^ ^]^ The RQC pathway has been well characterized in *S. cerevisiae*. Briefly, collided ribosomes during translation are monitored and ubiquitinated by an E3 ubiquitin ligase Hel2q, recruiting RQC complex to form a stable complex with 60S ribosomal subunits containing stalled polypeptides, which are ubiquitinated by Ltn1p and tagged for proteasome‐mediated degradation,^[^ ^29^ ^]^ and triggering ribosome subunit dissociation by the RQT complex.^[^ ^30^ ^]^ All the seven classically identified proteins involved in the RQC system were upregulated in *msp‐8* ^KO^ mutant (Figure 3C), indicating MSP‐8 deficiency may disrupt translational homeostasis or cause translational arrest. Furthermore, RQC‐related mutants, including NcRqc1, NcLtn1, and ZNF‐28, displayed increased antifungal sensitivity, suggesting MSP‐8 deletion may contribute to antifungal resistance via the upregulation of RQC‐related proteins (Figure S4F, Supporting Information). To assess the translational status in the MSP‐8 deleted strain, polysome profiling was performed and showed an unapparent change in the polysome/non‐polysome (P/Non‐P) ratio that reflects the general translation activity (Figure 3D; Figure S4G, Supporting Information).^[^ ^31^, ^32^ ^]^ However, the relative quantity of 80S monosomes in *msp‐8* ^KO^ mutant was 90.2% of that in WT (Figure 3D,E).

**Figure 4:**
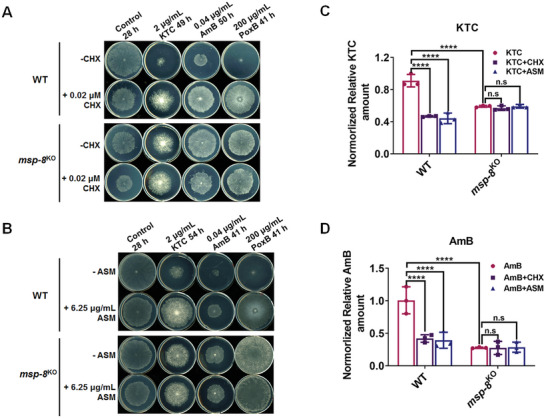
Deletion of MSP‐8 and translation inhibitors reduce both intracellular KTC accumulation and the membrane‐bound AmB content, leading to fungal multidrug resistance. A,B) Drug susceptibility test of the WT and *msp‐8* ^KO^ strains to designated concentration of different antifungals, either untreated or treated with 0.02 µM CHX (A), or 6.25 µg mL^−1^ ASM (B). Both CHX and ASM are protein translation inhibitors. C) Analysis of KTC accumulation in WT and *msp‐8* ^KO^ strains under KTC stress, and under KTC treatment combined with either CHX or ASC. D) Analysis of the membrane‐bound‐AmB content in WT and *msp‐8* ^KO^ strains under AmB stress, and under AmB treatment combined with either CHX or ASC. All the data are presented as mean ± SD. Statistical significance between each pair of groups was determined using two‐way ANOVA. Values with *p* < 0.0001 and *p* > 0.05 are marked with **** and n.s, respectively. Statistical values are as follows: *p_msp‐8_*_KO(KTC, KTC+CHX)_ = 0.7528, n = 3; *p_msp‐8_*_KO(KTC, KTC+ASM)_ = 0.9996, n = 3; *p_msp‐8_*_KO(AmB, AmB+CHX)_ = 0.9988, n = 3; *p_msp‐8_*_KO(AmB, AmB+ASM)_ = 0.9885, n = 3.

To examine translation's role in drug resistance, we added a low concentration of 0.02 µM CHX into Vogel's plates with or without the antifungal drugs. In WT, CHX suppressed mycelial growth and increased resistance to KTC, AmB, and PoxB (**Figure** **4**A), similar to the phenotypes observed in *msp‐8* ^KO^ on the Vogel's plate in the control and under KTC, AmB, PoxB stress. In contrast, *msp‐8* ^KO^ mutant showed only slight growth suppression and negligible changes in resistance to the corresponding antifungal drugs when supplemented with CHX. Similar effects were observed with ASM, another translational elongation inhibitor (Figure 4B), suggesting the mild inhibition of protein translation could increase multidrug resistance in *N. crassa*.

Based on our collective findings, MSP‐8 played a role in modulating the translation process, suggesting a connection between MSP‐8 and the translation machinery. Genes involved in translation, such as ribosome genes, their expression are generally downregulated under stresses and upregulated when translation is inhibited.^[^ ^33^ ^]^ The gene expression of *msp‐8* was observed to be downregulated under azole stress (Figure S4H, Supporting Information), exbiting a transcriptional response similar to that of ribosomal genes. Thus, it was interesting to investigate whether *msp‐8* can be transcriptionally modulated by translation inhibition. Using qRT‐PCR, we assessed the transcriptional levels of the *msp‐8* gene in the WT strain before and after treatment with either CHX or ASM. As expected, CHX treatment led to a significant induction of *msp‐8* expression, whereas ASM treatment resulted in a slight decrease in *msp‐8* transcriptional level (Figure S4I, Supporting Information). This discrepancy would be that CHX and ASM act on different stages of the translation process,^[^ ^34^ ^]^ resulting in different status of translation inhibition. Despite the difference, both treatments led to a transcriptional response of *msp‐8*, further indicating the association of MSP‐8 with translation.

### 2.4 MSP‐8 Deficiency Causes Decreased Intracellular ketoconazole (KTC) Accumulation and Membrane‐Bound AmB Content through Translation Disturbance

Using HPLC‐MS, we measured intracellular KTC accumulation and found it significantly reduced in the *msp‐8* ^KO^ strain, reaching only 65.0% of that in WT (Figure 4C). This decrease in KTC accumulation likely underlies the azole resistance in the *msp‐8* mutant. Notably, the deletion of MSP‐8 did not induce the expression of *cdr4* gene encoding the azole efflux pump (Figure S5A, Supporting Information), suggesting that reduced KTC accumulation in the *msp‐8* ^KO^ mutant was independent of CDR4‐mediated efflux and may instead result from impaired drug import.

When treated with the translational elongation inhibitors CHX (0.02 µm) or ASM (6.25 µg mL^−1^), the WT strain showed substantial reductions in KTC accumulation, with 51.3% and 48.5% of the pre‐treatment levels, respectively (Figure 4C). However, these treatments did not affect the KTC accumulation in the *msp‐8* ^KO^ mutant, consistent with the different responses of the WT and *msp‐8* ^KO^ strains to antifungal drugs with CHX or ASM in Figure 4A,B. Similarly, the content of membrane‐bound AmB in the *msp‐8* ^KO^ strain was only 27.6% of that in the WT strain (Figure 4D). It was reduced to 41.7% and 39.0% after CHX or ASM treatment in WT respectively, while remaining unchanged in the *msp‐8* ^KO^ strain (Figure 4D). These findings indicate that the deletion of *msp‐8* reduced intracellular KTC accumulation and membrane‐bound AmB content through translation disturbance.

### 2.5 MSP‐8 Deficiency Causes Down‐Regulation of Transport Proteins, Cell Wall Remodeling, and Reduced reactive oxygen species (ROS) Levels

**Figure 5:**
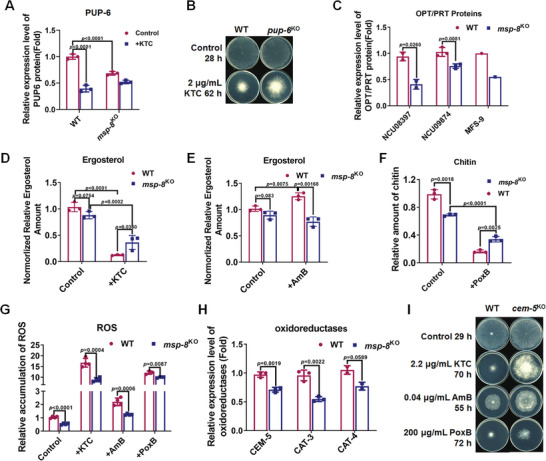
MSP‐8 deficiency leads to down‐regulation of transport proteins, remodeling of cell wall, and reduced ROS levels A) Relative expression of PUP‐6 protein in WT and *msp‐8* ^KO^ strains, with or without KTC treatment. The data are presented as mean ± SD with n = 3. Statistical significance between groups is determined by two‐way ANOVA. B) Drug susceptibility test of WT and *pup‐6* ^KO^ strains to KTC. C) Relative expression of oligopeptide/dipeptide transporters NCU08397, NCU09874, and MFS‐9 in WT and *msp‐8* ^KO^ strains. The data are presented as mean ± SD with n_NCU08397_ = 2 and n_NCU09874_ = 3. Statistical significance between groups is determined by the two‐tailed t‐test. D–F) Relative amounts of ergosterol (D,E) and chtin (F) in *msp‐8* ^KO^ and WT strains under control conditions and with KTC (D), AmB (E), or PoxB (F) treatment. These data are presented as mean ± SD with n = 3. Statistical significance between groups is determined by the two‐way ANOVA. G) Accmulation of ROS in the indicated strains under the untreated (control) or after treatment with KTC, AmB, and PoxB. The data are presented as mean ± SD with n = 3. Statistical significance between groups is determined by the two‐tailed *t*‐test. H) Relative expression of oxidoreductases CEM‐5, CAT‐3, and CAT‐4 in WT and *msp‐8* ^KO^ strains. The data are presented as mean ± SD with n_CEM‐5_ = 3, n_CAT‐3_ = 3, and n_CAT‐4_ = 2. Statistical significance between groups is determined by the two‐tailed *t*‐test. I) Drug susceptibility test of WT and *cem‐5* ^KO^ strains to KTC, AmB, and PoxB.

The decreased accumulation of KTC observed in the *msp‐8* mutant may be attributed to reduced drug import rather than CDR4‐mediated efflux. It has been reported that azole drugs are imported by facilitated diffusion in fungi.^[^ ^35^ ^]^ To identify the potential carrier or channel protein that contributed to KTC resistance, the proteome of the *msp‐8* mutant and the WT strain, with or without KTC treatment, were analyzed. As shown in **Figure** **5**A, compared to WT in the control, both KTC treatment and *msp‐8* deletion resulted in decreases in PUP‐6 expression, a purine‐cytosine permease, with 60.5% and 31.5% decreases respectively. Additionally, PUP‐6 deletion led to azole resistance in *N. crassa* (Figure 5B). It has been reported that the purine‐cytosine permeases are responsible for the active transport of 5‐flucytosine into the cell, which behave as a competitive inhibitor of fluconazole uptake transport, indicating purine‐cytosine permease may play roles in azole uptake.^[^ ^36^, ^37^ ^]^ These results indicated that decreased KTC accumulation in the *msp‐8* mutant may stem from impaired drug import through the downregulation of PUP‐6.

Proteomic analysis also identified downregulation of several oligopeptide/dipeptide transporters in *msp‐8* ^KO^ mutant, including NCU08397, NCU09874, and MFS‐9 (Figure 5C), the transcriptional expressions of which were found decreased in PoxB‐resistance evolved strains in WT under long‐term azole stress.^[^ ^23^ ^]^ Notably, the deletion of *mfs‐9* significantly increased resistance to PoxB in *N. crassa*. Since the chemical constitution of PoxB is similar to that of oligopeptide/dipeptide, the decreased expression of these peptide transporters probably contributes to decreased influx of PoxB transmembrane transport.^[^ ^23^, ^38^, ^39^ ^]^ Thus, *msp‐8* mutant increased resistance to PoxB possibly due to the lack or reduction of transport channels.

Given that ergosterol, an important cell membrane component and the target of the azoles, also directly binds to AmB,^[^ ^4^ ^]^ we quantified ergosterol amounts in *msp‐8* ^KO^ and WT under azole and AmB stress by HPLC‐MS. The ergosterol content was similar between WT and *msp‐8* ^KO^ in the control condition, and reduced to 87.7% and 59.5% relative to the corresponding strains respectively under KTC stress (Figure 5D). Under AmB stress, the WT strain increased ergosterol amounts to stabilize the membrane, whereas the *msp‐8* ^KO^ mutant showed no such increase (Figure 5E), likely correlating with reduced membrane bound AmB content. These observations indicated that the deletion of *msp‐8* enhanced KTC and AmB‐resistance by altering cell membrane components.

Transmission electron microscopy (TEM) revealed thinner inner cell wall and outer wall fibril were showed in *msp‐8* ^KO^ compared to that in WT (Figure S5B, Supporting Information). Cell wall component measurements further showed reduced chitin and β‐glucans contents in *msp‐8* ^KO^ (Figure 5F; Figure S5C, Supporting Information), consistent with lower transcript levels for genes involved in chitin biosynthesis (10 genes) and glucans biosynthesis (18 genes) (Figure S5D,E, Supporting Information). Under PoxB stress, *msp‐8* ^KO^ displayed increased inner and outer cell wall thickness, while WT thickened only the outer wall (Figure S5B, Supporting Information), suggesting that *msp‐8* ^KO^ was more adaptive under PoxB stress. Although chitin content decreased in both strains under PoxB stress, *msp‐8* ^KO^ showed less inhibition (50.8%) than WT (83.4%) (Figure 5F), indicating a more favorable adaptation in *msp‐8* ^KO^ under antifungal stress. Considering that the integrity of mycelial cell walls affects biofilm formation that is closely associated with antifungal resistance,^[^ ^40^ ^]^ we examined the biofilm formation capacity of *N. crassa* strains using crystal violet staining. Compared to WT and the *msp‐8* complementary strain (*msp‐8* ^KO^::*msp‐8* ^com^), deletion of *msp‐8* resulted in a slight reduction in biofilm formation capacity (Figure S5F, Supporting Information). This reduction was probably due to lower hyphal growth rate of *msp‐8* ^KO^ mutant in basic Vogel's medium (Figure 2B). We also examined the metabolic activity of the biofilms formed by *msp‐8* ^KO^, *msp‐8* ^KO^::*msp‐8* ^com^, and WT strains by 2,3‐bis(2‐Methoxy‐4‐nitro‐5‐sulfo‐phenyl)‐2H‐tetrazolium‐5‐carboxanilide (XTT) reduction assay. Under the control condition, the metabolic activity of the *msp‐8* ^KO^ biofilm was comparable to that of the WT. However, under stress induced by KTC, AmB, and PoxB, the *msp‐8* ^KO^ biofilm exhibited significantly increased relative metabolic activity (Figure S5G, Supporting Information). These results were consistent with the drug‐resistance phenotype of *msp‐8* ^KO^ mutant tested on the plates. Thus, the deletion of *msp‐8* modestly impaired biofilm formation; however, the formed biofilms demonstrated enhanced resistance to the tested antifungal agents.

Usually, antifungal drugs exert a harmful stress by binding to targets, as well as inducing cellular ROS levels, which are toxic to fungal cells.^[^ ^41^, ^42^ ^]^ ROS measurement showed the cellular ROS level in *msp‐8* ^KO^ is significantly lower than that in WT strain (Figure 5G), and the levels were induced in both *msp‐8* ^KO^ and WT strains under antifungal drugs treatment. However, the ROS contents in the *msp‐8* ^KO^ mutant were all significantly lower than those in WT strain under KTC, AmB, and PoxB treatment (Figure 5G), indicating a physiological environment conducive to antifungal tolerance. Proteomic analysis showed decreased expression of oxidative stress‐related proteins CEM‐5, CAT‐3, and CAT‐4 in *msp‐8* ^KO^ (Figure 5H). Drug susceptibility tests revealed that CEM‐5 deletion enhanced resistance to KTC, AmB, and PoxB (Figure 5I), suggesting that downregulated CEM‐5 contributed to multidrug resistance in the MSP‐8 mutant. Furthermore, decreased CAT‐3 and CAT‐4, enzymes involved in ROS clearance, maybe a feedback response to reduced ROS levels in *msp‐8* ^KO^.

## 3 Discussion

The molecular mechanisms underlying drug resistance, particularly those mediated by drug targets, efflux pumps, and their regulators are well‐characterized and represent classical causes of drug resistance. However, investigating additional drug resistance genes and their molecular functions remains essential for deepening our understanding of antifungal resistance and for informing the development of new drug targets. In this study, an evolved strain 2k‐6 with multidrug resistance to KTC and VRC (the azoles), AmB (the polyene), and PoxB (the chitin synthesis inhibitor) was rapidly obtained by experimental evolution using the ancestral strain *cdr4* ^KO^, the azole efflux pump deleted strain.Remarkably, the strain 2k‐6 acquired stable genetic resistance without detectable changes in the coding sequences and transcript levels of the azole target (ERG11) or other potential drug efflux pumps. Our analysis revealed a novel mutation in a DExD/H‐box helicase, MSP‐8, which primarily contributed to this multidrug resistance in strain 2k‐6. Furthermore, clinical *A. fumiguatus* strain 2707, harboring an amino acid substitution in Af*msp‐8* (F367Y), also displayed increased resistance to ITC and AmB, suggesting that mutations in MSP‐8 may underlie clinically relevant resistance in pathogenic fungi.

MSP‐8 is the homolog of yeast Ylr419wp, whose function is incompletely understood but known to be located in the cytoplasm and mitochondria according to high‐throughput studies in Saccharomyces Genome Database (SGD) (https://www.yeastgenome.org/locus/S000004411). We showed that MSP‐8 located in cytoplasm with a GFP fluorescent tracer and also colocalized with a mitotracker (Figure 3A). MSP‐8 interacted with the ribosomal subunit proteins such as CYH‐2, which is crucial for protein translation. A recent study determined the contacted region of Ylr419wp with the ribosome by analysis of interaction between Ylr419w‐HTP and the rRNA, and demonstrated that Ylr419wp was the structural homolog of mammalian translation initiation factor DHX29.^[^ ^43^ ^]^ Based on this, we propose that MSP‐8 primarily functions in the cytoplasm, where it modulates protein translation, while its mitochondrial role remains unclear. CHX arrests ribosomes by binding to the E site, preventing the E site tRNA from leaving the ribosome, thereby inhibiting elongation by maintaining them in a classical PRE state.^[^ ^26^ ^]^ The deletion of *msp‐8* may disrupt the binding of CHX to CYH‐2 and cause CHX resistance (Figure 4A). Another translation elongation inhibitor, ASM, maintains the ribosome in a rotated conformation distinct from that stabilized by CHX.^[^ ^34^ ^]^ ASM also enhances resistance to KTC, AmB, and PoxB in WT (Figure 4B), suggesting that the deletion of *msp‐8* may retard translation by affecting several ribosomal subunit sites, thereby increasing antifungal resistance in a manner akin to that of translation inhibitors. MSP‐8 deficiency inconspicuously changed the general translation activity by polysome profiling analysis. However, the 80S peak was significantly lower in the mutant than the WT (Figure 3D,E). The polysomes are generally equated with the translationally active mRNA pool, while monosomes have been often presumed to be translationally inactive.^[^ ^44^ ^]^ Heyer et al. redefined the translational status of 80S monosomes and highlighted the importance of 80S for the translation of highly regulated mRNAs.^[^ ^45^ ^]^ Therefore, MSP‐8 deficiency likely affects translation activity of some specified mRNA other than the overall translation landscape.

The Deletion of *msp‐8* was observed to activate the RQC pathway, as evidenced by the upregulation of seven classical RQC components that sense translation disturbance or ribosomal stalling in both eukaryotes and bacteria.^[^ ^28^, ^46^ ^]^ Notably, the E3‐ligase ZNF‐28 (Hel2p in yeast) acts as a central factor in the RQC pathway, connecting the sensing of collided ribosomes with the nascent polypeptide degradation.^[^ ^47^ ^]^ The RQC pathway has emerged as a central signal for translational stress, activating various cellular stress responses.^[^ ^27^, ^29^ ^]^ Thus, the deletion of *msp‐8* induces multidrug resistance by disrupting translation, offering insights into mechanisms underlying multidrug resistance. Usually, protein synthesis inhibition causes increased (m)TORC1 activity.^[^ ^48^ ^]^ While our data showed none of the gene/protein involved in the TOR signaling were differently expressed after deletion of *msp‐8* (File S5, Supporting Information). In addition, the fitness of *msp‐8* deletion mutant in the condition of mTor inhibitor (rapamycin) was assayed, and did not show obvious change compared to the WT strain (Figure S5I, Supporting Information). Thus, the deletion of MSP‐8 may not lead to the activation of the TOR signaling pathway, possibly because that MSP‐8 deficiency did not cause a global translation retardant as general protein synthesis inhibition. Moreover, this deficiency may be partially repaired under the regulation by the activation of RQC pathway, rendering it insufficient to activate the TOR pathway.

Both translation inhibitors CHX and ASM were shown to increase resistance to KTC and AmB by reducing intracellular drug accumulation, a phenotype similar to that caused by MSP‐8 deficiency. However, treating the *msp‐8* ^KO^ mutant with translation inhibitors did not significantly alter its drug sensitivity or intracellular drug accumulation. These findings underscore a correlation between translation regulation and fungal resistance, suggesting that translation control may modulate intracellular drug accumulation. This represents the first report, to our knowledge, of a helicase affecting multidrug resistance in fungi via translational regulation. Further investigation into the role of MSP‐8 in translatome, especially in terms of specific mRNA enriched in 80S monosomes separated in polysome profiling, would deepen our understanding of the mechanisms that induce multidrug resistance.

A recent study reported that CHX induced the expression of Cdrp1, an ABC‐transporter with a fast kinetic, which is a major contributor to pleiotropic drug resistance in *Candida glabrata*.^[^ ^49^ ^]^ Consistently, we found induction of the *cdr4* gene in *N. crassa* following CHX treatment; however, this did not significantly impact azole resistance, as intracellular KTC accumulation was also reduced in the *cdr4* ^KO^ mutant after CHX treatment (Figure S5H, Supporting Information). These findings suggest that reduced cellular KTC accumulation affected by translation inhibitors may be more closely linked to drug influx than efflux. Noël et al. (2003)^[^ ^36^ ^]^ demonstrated the activity of flucytosine‐uptake transport through kinetic transport studies, showing flucytosine resistance due to a defect in the purine‐cytosine permease, and predicted that flucytosine behaved as a competitive inhibitor of fluconazole uptake transport.^[^ ^36^ ^]^ We found that the expression of the purine‐cytosine permease PUP‐6 was decreased in WT under KTC stress by proteomic analysis, and the *pup‐6* ^KO^ mutant exhibited increased KTC resistance, indicating PUP‐6 in KTC uptake in *N. crassa*. The decreased PUP‐6 expression in the *msp‐8* ^KO^ mutant further suggests that *msp‐8* deletion may reduce cellular KTC accumulation by reducing KTC uptake.

CEM‐5 is a mitochondria‐related condensing enzyme with predicted oxidoreductase activity. Numerous studies have shown that mitochondrial dysfunction or downregulation of related genes can lead to fungal resistance,^[^ ^50^, ^51^, ^52^ ^]^ and oxidoreductases are also involved in many cellular stress response pathways, including oxidative stress induced by antifungal drugs.^[^ ^42^, ^53^ ^]^ While the functions of CEM‐5 and the homologs remain unclear, we speculated that CEM‐5 may play a role in the stress response of fungi to antifungal drugs by modulating cellular redox states.

Generally, antifungal drugs are not only bind the targets and inhibit the cell membrane and wall but also induce ROS production to damage cellular components.^[^ ^54^ ^]^ Many lines of evidence indicate that reduced cellular ROS accumulation has been associated with multidrug resistance in fungi.^[^ ^50^, ^55^ ^]^ In our study, ROS accumulations in the *msp‐8* ^KO^ mutant were consistently lower than those in the WT both in the presence and absence of drugs, suggesting that the deletion of *msp‐8* contribute to drug resistance may be through a reduction in baseline ROS levels.

The biofilm has been widely recognized as a state affords microorganism with multidrug resistance,^[^ ^14^ ^]^ and Hsp90 orchestrates stress response signaling governing fungal drug resistance.^[^ ^56^, ^57^ ^]^ Thus, we also examined the biofilm formation capacity (Figure S5F, Supporting Information) and analyzed the gene and protein expression levels associated with the HSP90‐calcineurin regulatory pathway (File S5, Supporting Information). None of these were involved in the resistant mechanism conferred by MSP‐8. In addition, the fitness of *msp‐8* deletion mutant under the condition treated with the HSP90 inhibitor geldanamycin was assayed. The mutant exhibited no significant difference in fitness compared to the WT strain (Figure S5I, Supporting Information). These findings suggest that drug resistance conferred by the deletion of *msp‐8* is not mediated through activation of the HSP90 pathway.

Our results showed that the *msp‐8* mutation confers multidrug resistance to the azoles, AmB, and PoxB, and reduced mycelial growth rate. These data suggest that MSP‐8 is necessary for normal fungal growth. Previous studies have shown that ribosomal mutations affecting ribosome assembly and causing large‐scale transcriptomic and proteomic changes have a fitness cost in antibiotic‐free medium and promote the evolution of high‐level resistance in a multidrug environment.^[^ ^58^ ^]^ Ribosome biogenesis and protein synthesis are fundamental rate‐limiting steps for cell growth and proliferation.^[^ ^59^, ^60^ ^]^ The involvement of ribosomal protein genes in translation and the direct relation of translation rates to exponential growth rates ensure that fitness effects are directly correlated to the quality and quantity of available ribosomes.^[^ ^61^, ^62^ ^]^ Therefore, the fitness cost in an antifungal drug‐free medium may result from reduced available ribosomes and the delayed translation in the *msp‐8* mutant. Fitness cost of resistance are universal in fungi, bacteria, and cancer cells,^[^ ^63^ ^]^ yet the underlying mechanisms in the absence of the drug are complex and remain poorly understood. We speculated that MSP‐8 deficiency confers multidrug resistance with a fitness cost by impairing ribosome quality and availability, supporting a link between ribosomal proteins and fitness costs.

## 4 Conclusion

In summary, we identified *msp‐8* as a novel multidrug‐resistance gene encoding a helicase in fungi, and uncovered a previously uncharacterized multidrug‐resistance mechanism where dysfunction of helicase MSP‐8 leads to suppressed protein translation, thereby conferring multidrug resistance in fungi.

## 5 Experimental Section

### Strains and Media

All the strains used in this study are listed in Table S1 (Supporting Information). Point mutation or complementation of *msp‐8* in *N. crassa* was introduced via overlapping PCR products with chlorimuron‐ethy resistance cassettes and 5′ and 3′ flanking sequences (1–1.5 kb) of the coding genes. The deletion construct was introduced into the relevant recipient strain by homologous recombination method. *N. crassa* and *F. verticillioides* transformants were selected on medium supplemented with hygromycin B (150 µg mL^−1^), and those of parental strain CEA17 of *A. fumigatus* were selected with MM medium without uracil or uridine supplemention. The primers are listed in Table S2 (Supporting Information). The *N. crassa* wild‐type (FGSC#4200 [WT], *mat* a; FGSC#2225, *mat* A) and the constructed strains were cultured on Vogel's medium. Sorbose plates were used for electroporation transformation. The synthetic cross medium was for cross. The media and culture method were referenced to Perkins 2006.^[^ ^64^ ^]^ The *A. fumigatus* strains CEA17, ATCC was cultured on CM medium supplemented with 0.5 g L^−1^ uracil and 1.22 g/L uridine. Liquid YGU medium (YG medium and supplemented with 0.5 g L^−1^ uracil and 1.22 g L^−1^ uridine) was for spore germination. MM medium was for transformant screening. The media and culture method were referenced to Li et al. 2020.^[^ ^50^ ^]^ The *F. verticillioides* (FGSC#7600) and the knockout mutants strain were cultured on PDA plates (200 g/L potato, 2% glucose, and added with 1.5% agar). Liquid YPD medium (2% glucose, 2% peptone, 1% yeast extract, and 1.5% agar) was for spore germination and mycelial growth. All cultures were grown at 28 °C. The information of the genes was referenced to FungiDB (https://fungidb.org/fungidb/app) and FGSC database (https://www.fgsc.net/).

### Experimental Evolution of Azole Resistance

Experimental evolution in *N. crassa* was described previously.^[^ ^23^ ^]^ Briefly, ten independent populations of *N. crassa* were developed from a single azole‐susceptible isolate of the *cdr4* ^KO^ strain. The lineage K1, K3, and K4 were evolved by culturing *cdr4* ^KO^ on plates with a stepwise increased concentration of ketoconazole (KTC), while the lineages V1, V3, and V4 were evolved with voriconazole (VRC); and the lineage C1, C2, C3, and C4 were evolved with no drug (Data about lineage C4 was shown in this study). The experimental evolutionary populations were propagated for nearly 30 transfers (≈1 week incubation before each transfer).

### Drug Susceptibility Test

KTC, VRC, and amphotericin B were dissolved in dimethyl sulphoxide (DMSO), and Polyoxin B (PoxB, Baoli'an, Tokyo, Japan) was dissolved in ddH_2_O. These drug solutions were then sterilized by filtration. The drug susceptibility test for *N. crassa* was referenced to the previous studies.^[^ ^23^, ^65^ ^]^ For *A. fumigatus*, two microliter aliquots of conidial suspension with gradient dilution (10^7^, 10^6^, 10^5^ /mL) were incubated on CM plate with or without the above drugs at 37 °C for the indicated time. The images and diameters of colonies were documented after a certain time of incubation. For *F. verticillioides*, two microliter aliquots of conidial suspension with gradient dilution (10^7^, 10^6^, 10^5^, 10^4^/mL) were incubated on PDA plate with or without the above drugs at 28 °C for the indicated time.

The minimum inhibitory concentration (MIC) values of drugs were determined in 96‐well plates referring to Clinical and Laboratory Standards Institute (CLSI) broth microdilution method (M38‐A2 document) with some modifications. The liquid Vogel's medium was used to dilute the drugs and spores at designated concentrations. The 96‐well plates were incubated at 28 °C for 40 h and followed by microscopic observation.

### RNA Extraction, RT‐PCR, and RNA‐seq Analysis

Sample preparation and RNA extraction were carried out as described previously.^[^ ^23^ ^]^ Briefly, 15 pieces shredded circular mycelial mat (Φ = 2 mm) were transferred to Vogel's medium and incubated at 28 °C with shaking at 200 rpm for 24 h. Mycelium was then harvested and freshly frozen in liquid nitrogen for RNA extraction. To assess transcriptional changes under drugs treatment, the samples were added with the antifungal drug (KTC, AmB, or PoxB) after 12 h of incubation, and harvested for RNA extraction after another 12 h. RNA extraction and inversion were carried out as described previously.^[^ ^65^ ^]^ Gene‐specific primers are shown in Table S3 (Supporting Information).

Sequencing the transcriptome profiles of the WT and *msp‐8* ^KO^, either treated with KTC, AmB, PoxB or left untreated, were sequenced on NBSEQ platform by Bgi Genomics Co., Ltd (Shenzhen, China). After filtering by software SOAPnuke (v1.4.0), each sample produces an average of 6.38 Gb of clean data. The average comparison rate between samples and the referenced genomes of *N crassa* OR74A is 84.15%. Gene expression levels of each sample were calculated by RSEM (v1.2.8).^[^ ^66^ ^]^ Each treatment of the sample contained three biological replicates. The raw data of RNA‐seq has been deposited in the Short Read Archive (SRA) database, with accession number PRJNA1142505 (SRR31305185, SRR31305186, SRR31305187, SRR31305188, SRR31305189, SRR31305190).

### Extraction and Analysis of Intracellular Ergosterol, KTC, and AmB

Samples for ergosterol and KTC analysis were prepared as for RNA extraction. After harvest, the mycelia were freeze‐dried at ‐80 °C and ground into a fine powder. Intracellular ergosterol, KTC, and AmB were extracted with chloroform, and ergosterol and KTC were analyzed by HPLC‐MS (AGILENT 1200HPLC) as reported previously,^[^ ^65^ ^]^ using ergosterol and KTC as standards. AmB was extracted and dissolved with methanol: DMSO (84:16) as reported previous.^[^ ^67^ ^]^ AmB were analyzed by Ion Trap LC‐MS, using natamycin as standards. This experiment was performed independently three times. The software of Qualitative Analysis in B.07.00. was used for the analysis of ergosterol and KTC. The Analyst software was used for the analysis of AmB.

### Measurement of rhodamine 6G (R6G) Uptake and Glucose‐Induced Efflux Abilities

The intracellular concentration of R6G was evaluated as described previously^[^ ^68^ ^]^ with slight modifications. Briefly, approximately 5×10^6^ fresh conidia were grown in Vogel's medium at 28 °C until conidia began to germinate, and then washed and suspended in PBS, to which R6G was added at a final concentration of 10 µm. After incubation at 28 °C for 1 h, the samples were then washed with PBS, and a total of 30 000 events of each sample were captured using flow cytometer (BD influx), and the fluorescence of R6G in samples was measured. To assess energy‐dependent R6G efflux, the samples were suspended in PBS containing 0.5 m glucose and incubated for 15 and 30 min at 28 °C respectively, and followed by measuring the fluorescence intensity of remaining intracellular R6G.

### Extraction and Analysis of Chitin and β‐Glucans

Samples were harvested and ground into a fine powder for ergosterol preparation. The chitin and β‐glucans were isolated and determined as described previously^[^ ^69^ ^]^ with some modifications. Briefly, 10 mg mycelial powder was treated with 3% NaOH at 75 °C for 1 hour and divided into alkali‐soluble (AS) and alkali‐insoluble (AI) part. AI riched in chitin and β‐glucans were washed three times with distilled water and then treated with 6 N HCl at 100 °C for 2 h. After removal of HCl by evaporation, the residues were dissolved in 200 µL distilled water. The amount of β‐glucans was determined by measuring the glucan content with phenol/sulfuric acid. The amount of chitin was determined by measuring the N‐acetylglucosamine.

### Yeast Two‐Hybrid (Y2H) High Throughput Screening

A cDNA library of *N. crassa* for Y2H was constructed by Gateway recombination technology. Using the MSP‐8 CDS constructed on the pGBKT7 vector as baFit (no self‐activation), the cDNA library of *N. crassa* was screened. After screening on SD/‐Trp/‐Leu (SD‐2) or the selective medium SD/‐Trp/‐Leu/‐His/‐Ade (SD‐4), DNA sequencing, and BLAST comparison analysis of positive clones, the proteins that interact with pGBKT7‐MSP‐8 were identified. To thoroughly screen the interacting proteins, all positive clones of the yeast screening library were scraped with 2×YPDA liquid, centrifuged and collected for colony PCR, and the PCR products were subjected to NGS sequencing. The sequence data have been deposited in the Short Read Archive (SRA) database, with accession number SRR30091986.

### Bulked Segregant Analysis (BSA) and Cleaved Amplified Polymorphic Sequence (CAPS) Analysis

The BSA and CAPS analysis for location of mutation site coupled with multidrug resistance was conducted as described previously^[^ ^24^ ^]^ with slight modifications. Briefly, the evolved strain 2k‐6 (Oak Ridge mat *a*) and FGSC#2225 (Mauriceville‐1c mat *A*) were crossed and 100 progenies were picked. These were divided into multidrug‐resistant group and sensitive group according to drug susceptibility tests and each individual progeny was used as a source of gDNA. The independently extracted gDNA samples were added into equimolar mixtures composed of DNA pool in each group. CAPS assay was performed on markers using primer pairs listed in Table S4 (Supporting Information), and map ratio was calculated according to the study.^[^ ^24^ ^]^

### The Whole Genome Resequencing

Qualified DNA sample(s) were constructed BS library. Purified DNA sample, such as genomic DNA is sheared into smaller fragments with a desired size by Covaris S/E210 or Bioruptor first. Then the overhangs resulting from fragmentation were converted into blunt ends by using T4 DNA polymerase, Klenow Fragment, and T4 Polynucleotide Kinase. After adding an “A” base to the 3′ end of the blunt phosphorylated DNA fragments, adapters are ligated to the ends of the DNA fragments. The desired fragments were purified through gel‐electrophoresis, then selectively enriched and amplified by PCR. The index tag was introduced into the adapter at the PCR stage as appropriate and a library quality test was done. At last, the qualified BS library was used for sequencing. In order to obtain more accurate and reliable results in subsequent bioinformatics analysis, the raw data were further treated: 1) remove reads with a certain proportion of low quality (≤20) bases (40% as default). 2) Remove reads with a certain proportion of Ns (40% as default). 3) Remove adapter contamination. 4) Remove duplication contamination. By using software BWA (Burrows‐Wheeler Aligner) and then mapping the clean data to the reference, the bam results were obtained. To ensure the accuracy of mutation detection, the study took the analysis as GATK optimal variation detection and analysis process (Genome Analysis Toolkit). Based on the comparison results, the evaluation indexes such as the depth, coverage, and ratio of each sample were counted. Sequence data of the genomes have been deposited in the Short Read Archive (SRA) database, with accession number PRJNA1142505 (SRR30091648 and SRR30091649)

### TMT‐Labeled Proteomic Profiling

Samples for proteomic profiling analysis were prepared as for RNA extraction. Samples were ground into a fine powder in liquid nitrogen and lysed at 4 °C with lysis buffer supplemented with protease inhibitors (1% TritonX‐100, 10 mm dithiothreitol, and 1% protease inhibitor cocktail, 50 µM PR‐619, 3 µm TSA, 50 mm NAM, 2 mm EDTA, and 1% phosphatase inhibitor for phosphorylation). Proteins were precipitated by ammonium sulfate‐saturated methanol and incubated at −20 °C for at least 6 h and washed with ice‐cold methanol, followed by washing in ice‐cold acetone for three times. The protein was redissolved in 8 m urea and the protein concentration was determined with BCA kit according to the manufacturer's instructions. After trypsin digestion, each channel of tryptic peptide was labeled with their respective TMT reagent (based on manufacturers protocol, ThermoFisher Scientific), and incubated for 2 hours at room temperature. Samples were quenched by adding 5% hydroxylamine, desalted with Strata X C18 SPE column (Phenomenex), dried by vacuum centrifugation, and then fractionated into fractions by high pH reverse‐phase HPLC using Agilent 300 Extend C18 column (5 µm particles, 4.6 mm ID, 250 mm length). For LC‐MS/MS analysis, the tryptic peptides were dissolved in solvent A, and directly loaded onto a home‐made reversed‐phase analytical column (25 cm length, 100 µm i.d.). Peptides were separated with the following gradient: 0–4 min, 7–10%B; 4–53 min, 10%‐32%B;53–57 min, 32–80%B; 57–60 min, 80%B, at a constant flow rate of 500 nl/minon a EASY‐nLC 1200 UPLC system (ThermoFisher Scientific). The separated peptides were analyzed in Orbitrap Exploris 480 with a nano‐electrospray ion source. The resulting MS/MS data were processed using MaxQuant search engine (v.1.6.15.0). Tandem mass spectra were searched against Blast *N. crassa* strain ATCC_24698_367110_PR_20230529.fasta (10256 entries) concatenated with reverse decoy and contaminants database. Sequence data of the proteomics have been deposited in the iProX database, with accession number PXD054574. The Gene Ontology enrichment analysis of the differently expressed proteins was used DAVID (https://david.ncifcrf.gov/).

### Co‐IP Assay Immunoprecipitation and Immunoblotting

3×Flag‐CYH‐2 and MSP‐8‐5×MYC were co‐expressed in the *msp‐8* ^KO^ mutant. For Co‐IP experiments, mycelia were ground into a fine powder after fast freezing in liquid nitrogen in 1.5 ml EP tube and lysed on ice for 5 min in lysis buffer with protease inhibitors (10 mm Tris [pH 7.5], 150 mM NaCl, 0.5 mM EDTA, 0.5% Nonidet P‐40, 0.5 mM PMSF, 1 µg mL^−1^ leupeptin and 1 µg mL^−1^ pepstatin A). 5% supernatant lysate was taken as input and the rest was purified with Myc‐Trap agarose kit. Protein Myc‐Trap agarose‐enriched complexes were resolved on SDS‐PAGE gels and transferred onto PVDF membranes. Protein were detected with mouse anti‐FLAG monoclonal antibody (1: 1000 dilution) or mouse MYC tag monoclonal antibody (1:2000 dilution). An HRP‐conjugated anti‐mouse antibody (1:5000 dilutions) was used as the secondary antibody. The protein signals were visualized by ECL exposure.

### Polysome Profiling

Polysome preparation was performed according to previously described protocols^[^ ^26^ ^]^ with slight modifications. Briefly, 1 × 10^8^ fresh spores were inoculated in 100 mL Vogel's medium and germinated to 30 µm hypha, then treated with CHX for 10 minutes before harvest. The harvested samples were freshly frozen in liquid nitrogen and ground into power, then lysed in 500 µL of cold lysis buffer (QingZebio, Guangzhou). The lysate was centrifuged at 4 °C for 3 min at 13 000 g to pellet cell debris. The cytoplasmic lysates were separated in a linear 10–45% [w/v] sucrose gradient. Fractions of 100 µL of volume, were then collected monitoring the absorbance at 260 nm with the Full‐automatic component separator (Biocamp).

### Biofilm Quantification and 2,3‐bis(2‐Methoxy‐4‐nitro‐5‐sulfo‐phenyl)‐2H‐tetrazolium‐5‐ carboxanilide (XTT) Reduction Assay

Biofilm biomass and XTT assay were assessed according to previously described protocols^[^ ^70^ ^]^ with slight modifications. Briefly, 150 µL fresh spores (1 × 10^5^/mL) were inoculated in 96‐well microtiter plate in 28 °C for 24 h, followed by washing the biofilm three times with PBS. Then 1% (w/v) crystal violet solution was added, followed by washing the stained biofilm three times with water until excess stain was removed. 95% ethanol was added and transferred to a clean 96‐well microtiter plate and the A600 was read.

After the fresh spores were incubated for 12 hours, the culture medium was removed and replaced with Vogel's media containing appropriate concentrations of antifungal drugs. The culture was incubated for another 12 hours before adding a volume of solution containing XTT (0.5 mg mL^−1^) and menadione (10 µm). After incubation for another 2–3 hours, the reaction solution was transferred to a clean 96‐well plate to measure the absorbance at 460 nm.

### Measurements of ROS Contents

For ROS content measurement, fresh spores were harvested from 7‐day‐old cultured slant before loaded with diluted DCFH‐DA. The protocol for measuring ROS was referenced to the Assay Kit (S0033M, Beyotime).

### Statistical Analysis

Statistical analysis was performed by GraphPad Prism v.8. Two‐tailed unpaired Student's *t*‐test was used to compare the relative amount of the inhibitory rate, the ratios of 80S/Non‐P and P/Non‐P, the relative expression levels of genes, and the relative accumulation of ROS between two groups. The two‐way ANOVA method was used to evaluate the significance between two independent variables under several conditions, eg normalized relative KTC and AmB amount, and the mean fluorescence intensity or transcript levels between two groups. A two‐sided P< 0.05 was considered significant. The data are shown as means ± SD from three or more independent experiments.

## Conflict of Interest

The authors declare no conflict of interest.

## Supporting information



Supporting Information

Supporting Information

## Data Availability

The sequence data of the proteomics that support the findings of this study are openly available in iProX at https://www.iprox.cn/page/project.html?id=IPX0009396000, with accession number PXD054574. The sequence data of RNA‐seq, Y2H high throughput, and the whole genome resequencing have been deposited in the Short Read Archive (SRA) database, with accession number PRJNA1142505 (https://www.ncbi.nlm.nih.gov/sra/PRJNA1142505).
