# Supplementary material for: Suppressed Protein Translation Caused by MSP‐8 Deficiency Determines Fungal Multidrug Resistance with Fitness Cost
Source: Adv Sci (Weinh). 2024 Dec 16;12(6):2412514. doi: 10.1002/advs.202412514 (PMC11809369; doi:10.1002/advs.202412514)
Supplement: Supplementary file 1 — Supporting Information [file ADVS-12-2412514-s001.docx]

Supporting Information

Suppressed Protein Translation Caused by MSP-8 Deficiency Determines Fungal Multidrug Resistance with Fitness Cost

Mi Zhou, Pengju Yu, Chengcheng Hu, Wenxia Fang, Cheng Jin, Shaojie Li,^*^ and Xianyun Sun^*^

**
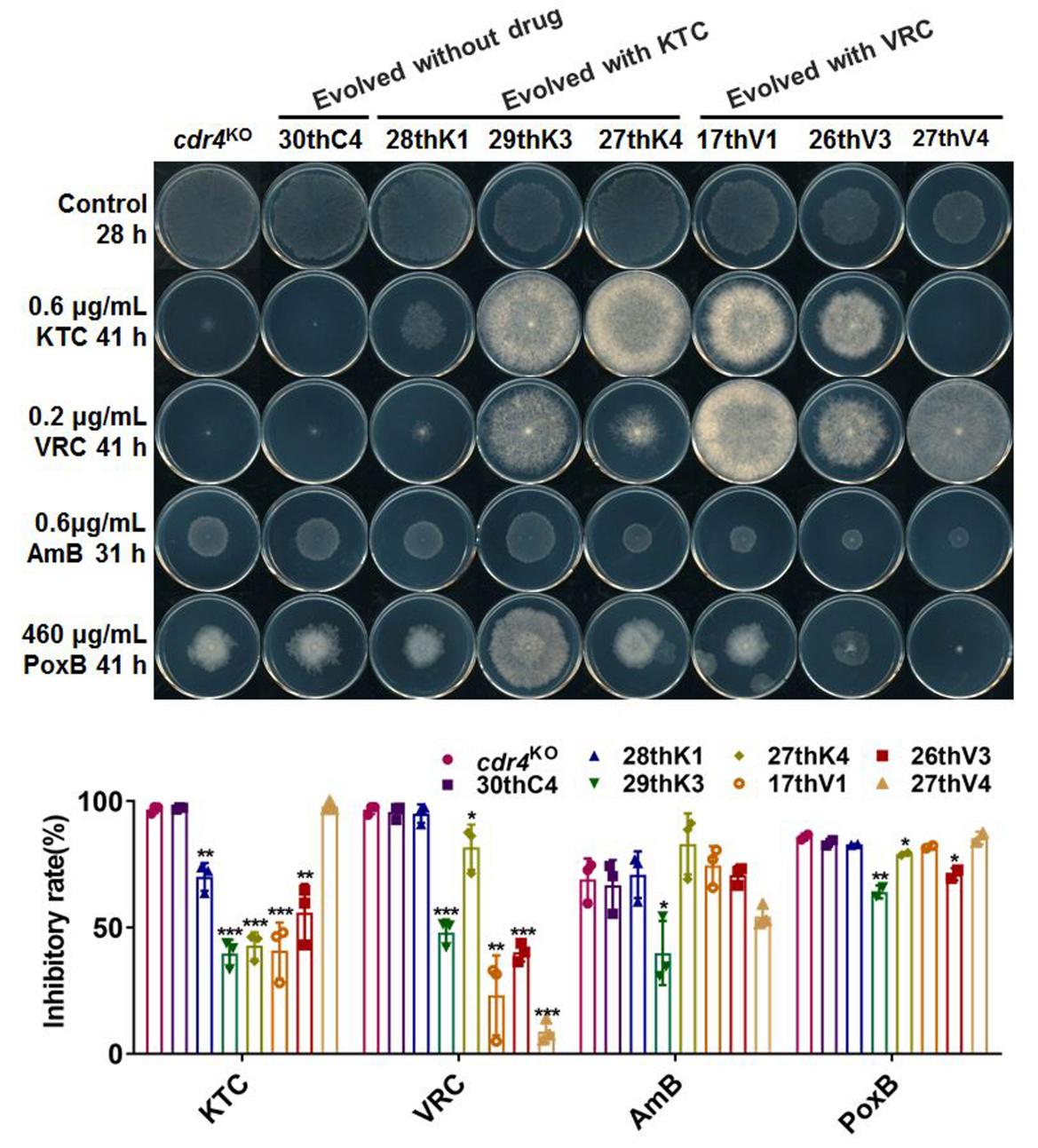
**

**Figure S1. Acquired antifungal resistance in *N. crassa* *cdr4* knockout mutant (*cdr4*^KO^) under azole stress.** The evolved populations are tested for susceptibility to KTC, VRC, AmB and PoxB, as depicted in the figure. Relative growth inhibition rates are calculated based on colony diameters of the strains grown on plates with and without antifungals at the indicated growth time. The data are presented as mean ± SD. Statistical significance between the evolved strains and the ancestor *cdr4*^KO^ strain were determined by the two-tailed t-test. Values with *p* < 0.001, 0.001 < *p* < 0.01, and 0.01 < *p* < 0.05 are marked with***, ** and *, respectively. Statistical values are as follows: *p_KTC(28thK1, cdr4_*_KO_*_)_*= 0.00124403, n=3; *p_KTC(29thK3, cdr4_*_KO_*_)_*= 6.450551e-005, n=3; *p_KTC(27thK4, cdr4_*_KO_*_)_=*7.123587e-005, n=3; *p_KTC(17thV1, cdr4_*_KO_*_)_*=0.000964257, n=3; *p_KTC(26thV3, cdr4_*_KO_*_)_*=0.0034571, n=3; *p_VRC(29thK3, cdr4_*_KO_*_)_*=9.717021e-005 , n=3; *p_VRC(27thK4, cdr4_*_KO_*_)_=*0.0475644, n=3; *p_VRC(17thV1, cdr4_*_KO_*_)_*= 0.00132101, n=3; *p_VRC(26thV3, cdr4_*_KO_*_)_*= 1.742725e-005, n=3; *p_VRC(27thV4, cdr4_*_KO_*_)_*= 5.462532e-006 , n=3;*p_AmB(29thK3, cdr4_*_KO_*_)_*=0.0289826 , n=3; *p_PoxB(29thK3, cdr4_*_KO_*_)_*=0.0089119 , n=2; *p_PoxB(27thK4, cdr4_*_KO_*_)_=*0.0250854, n=2; *p_PoxB(26thV3, cdr4_*_KO_*_)_*= 0.0169706, n=2. The abbreviation for the antifungal drugs in this figure and the manuscript are as follows: KTC (ketoconazole), VRC (voriconazole), PoxB (polyoxin B), AmB (amphotericin B).

**
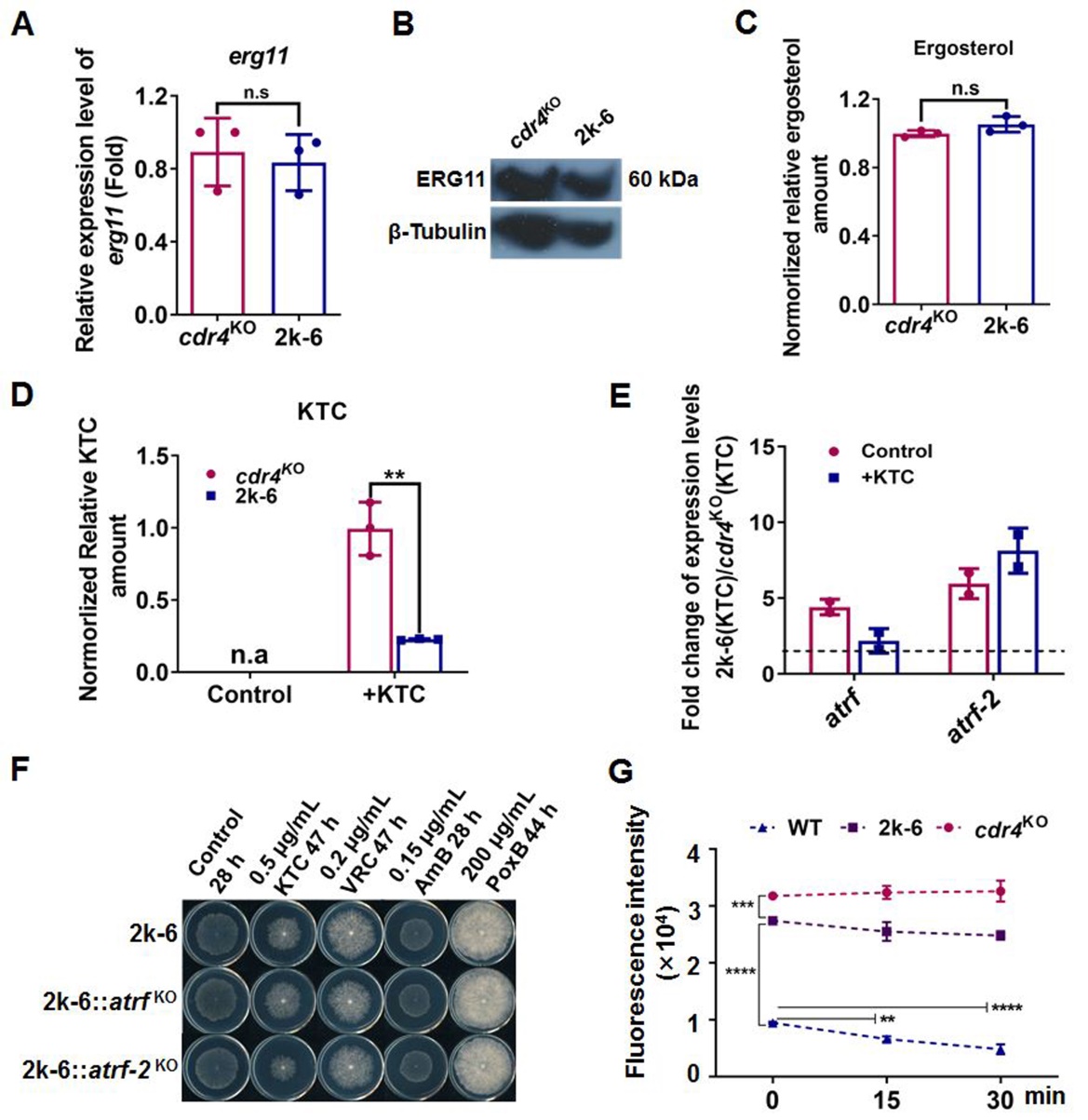
**

**Figure S2. Adaptive evolution to the azoles in strain 2k-6 is uncorrelated to the target nor the efflux pumps. (A)** Transcript levels of *erg11* gene in 2k-6 and *cdr4*^KO^ strains are measured by qPCR, calculated using the 2^-ΔΔCt^ method and normalized to *β-tubulin*. **(B)** Protein expression levels of ERG11 in 2k-6 and *cdr4*^KO^ strains are analyzed by western blotting and normalized to β-TUBULIN. **(C, D)** Accumulations of ergosterol (C) and KTC (D) in 2k-6 and *cdr4*^KO^ strains are measured by HPLC-MS. Statistical significance in (A, C and D) is determined by the two-tailed t-test and marked as n.s (*p* ≥ 0.05), ** (0.001< *p* < 0.01). Statistical values are as follows: *p*_erg11(cdr4KO, 2k-6)_=0.6992, n = 3. *p*_ergosterol(cdr4KO, 2k-6)_=0.1258, n = 3. *p*_KTC(cdr4KO, 2k-6)_=0.001974, n = 3. **(E)** Fold change between 2k-6/*cdr4*^KO^ and 2k-6(+KTC)/*cdr4*^KO^(+KTC) for the transcript levels of *atrf* and *atrf-2*. **(F)** Drug susceptibility test of the 2k-6::*atrf*^KO^ and 2k-6::*atrf-2*^KO^ mutants to different antifungals at designated concentrations. **(G)** Accumulation of rhodamine 6G (R6G) is detected by fluorescence-activated cell sorting (FACS) in 2k-6, *cdr4*^KO^ and WT strains. The X-axis presents the processing time of 0.1 M glucose, with fluorescence intensity at 0 min reflecting the R6G-intake amount in the indicated strains before glucose treatment. All the data are presented as mean ± SD. Statistical significance was calculated by Two-way ANOVA method and marked as **(*p*<0.01), ***(*p*<0.001), ****(*p*<0.0001). Statistical values are as follows: *p*_0(cdr4KO, 2k-6)_ = 0.0001, n = 3, *p*_WT(0, 15)_ = 0.0079, n = 3.

**
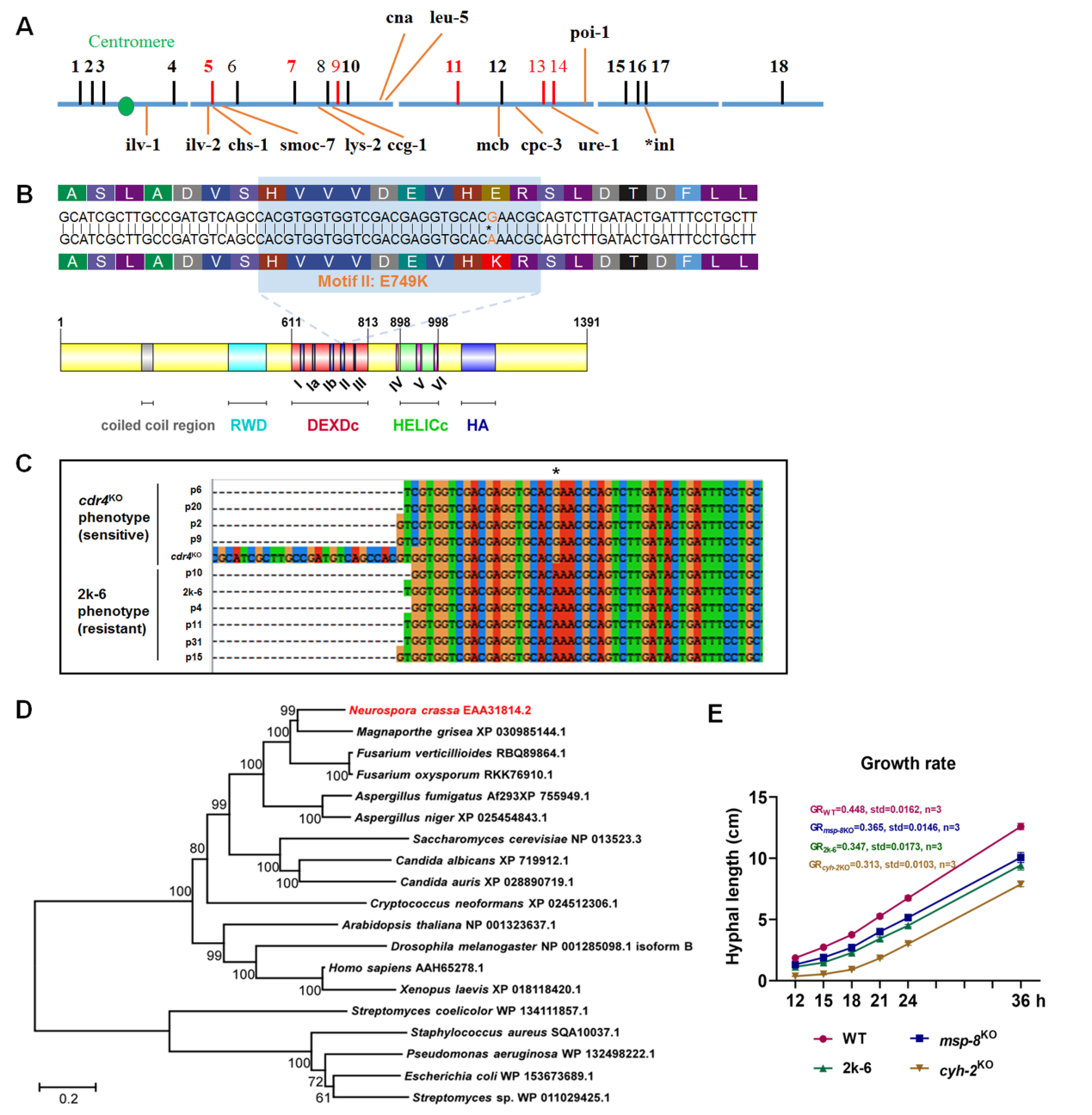
**

**Figure S3.** (**A**) Schematic representation of the CAPS markers and genetic makers on linkage group V (LGV). The numbers marked in red were CAPS makers genetically closed to the mutation from *ilv-2* (position 1828369) to *poi-1* (position 3397430). (**B**) Domain organization of the MSP-8 protein, highlighting the conserved RWD, DEXDc, HELICc and HA2 domains, which are characteristic of helicases. The amino acid substitution from glutamate (E) to lysine (K) at position 749 in the DEXDc is indicated. (**C**) Fragment sequencing of the *msp-8* gene in randomly selected progenies (p2, p6, p9, p20, p4, p10, p11, p15 and p31) from the 2k-6 and FGSC#2225 strains, together with *cdr4*^KO^ and 2k-6. (**D**) Phylogenic analysis of fungal MSP-8 homologs are identified through NCBI (National Center for Biotechnology Information) BLAST searches. The phylogenic tree is constructed using the Neighbor-Joining method in MEGA7 software. Bootstrap support for internal branches is based on 2000 replications. (**E**) Growth rates of the WT, *msp-8*^KO^, 2k-6, and *cyh-2*^KO^ strains, calculated based on colony diameters of the strains grown on plates during the indicated growth time (12-36 h). The data are presented as mean ± SD with n=3.

**
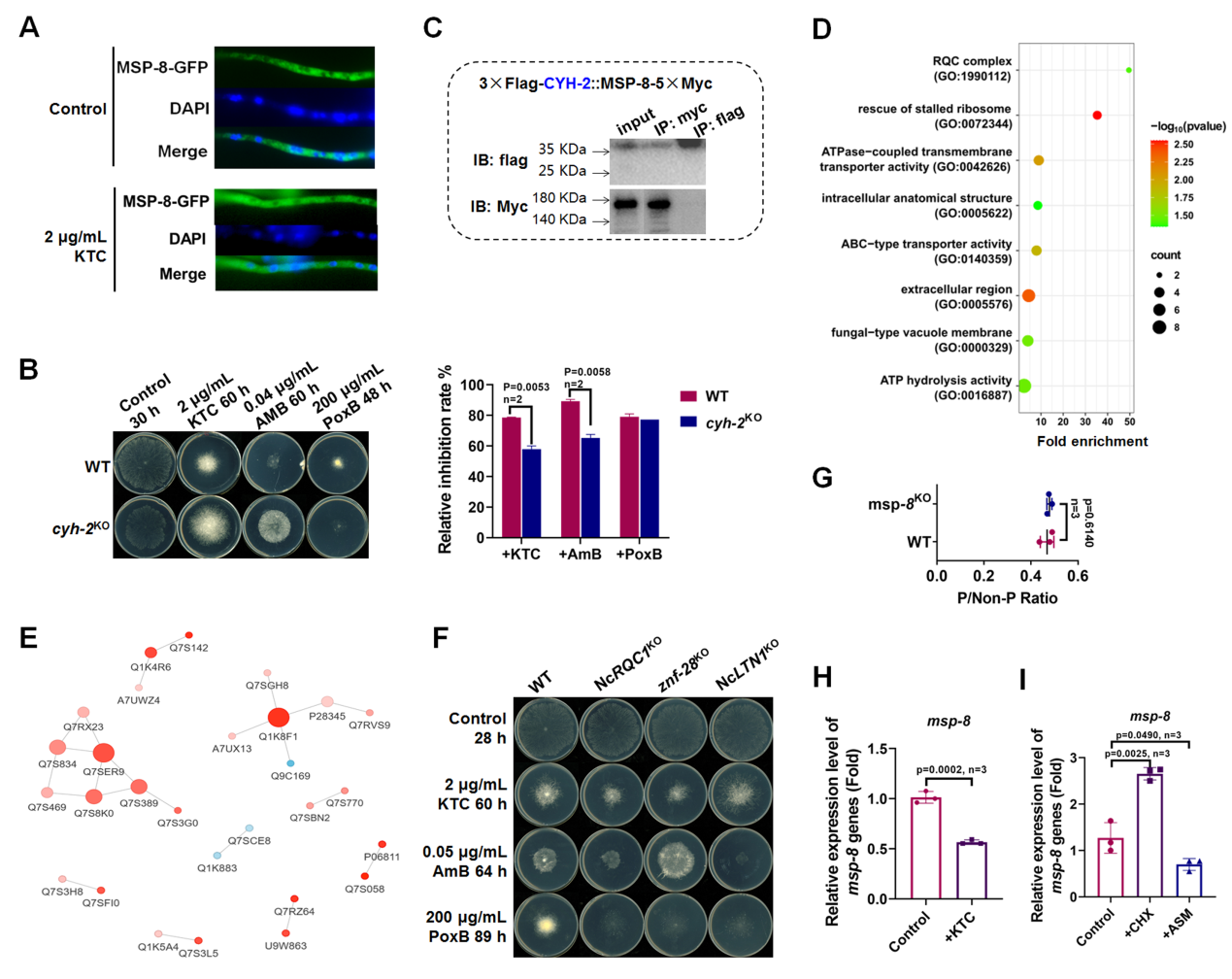
**

**Figure S4. (A)** Expression of the MSP-8-GFP fusion protein is in the *msp-8* mutant. After spore germination, hyphae of the appropriate length (5-8 h) are treated with or without 2 μg/mL KTC for 1 hour, then stained with DAPI for 20 min, and observed under a fluorescence microscope. (**B**) Drug susceptibility test of the *cyh-2* mutants to designated concentrations of various antifungals. Relative growth inhibition rates are calculated based on colony diameters of the strains grown on plates with and without antifungals at the indicated growth time. **(C)** Co-IP assay validating the interaction between MSP-8 and CYH-2. Detail method is provided as the Material and Methods section. **(D)** Gene ontology (GO) analysis of the differently expressed proteins between *msp-8*^KO^ and wild-type (WT) strains. Enriched GO terms are presented along with hypergeometric p-value, fold enrichment (X-axis) and gene count (indicated by circle size). (**E**) Protein-protein interaction (PPI) network of differentially expressed proteins, retrieved from the STRING database, with an interaction confidence score > 0.7. Source data of the differently expressed proteins between *msp-8^KO^* and WT are provided in File S4. **(F)** Drug susceptibility test of mutants of the RQC system proteins to designated concentration of various antifungals. **(G)** Polysome profiles obtained from WT and *msp-8*^KO^ strains, along with corresponding quantification of polysome to non-polysome (P/Non-P) ratios. (**H**) Relative expression level of the *msp-8* gene in WT strain under KTC stress, as determined by RNA-seq analysis. (**I**) Relative expression levels of the *msp-8* gene in WT strain before and after treatment of CHX or ASM, as determined by qRT-PCR. All the data are presented as mean ± SD. Statistical significance for (**B**), (**G**), (**H**), and (**I**) was determined by the two-tailed t-test.

**
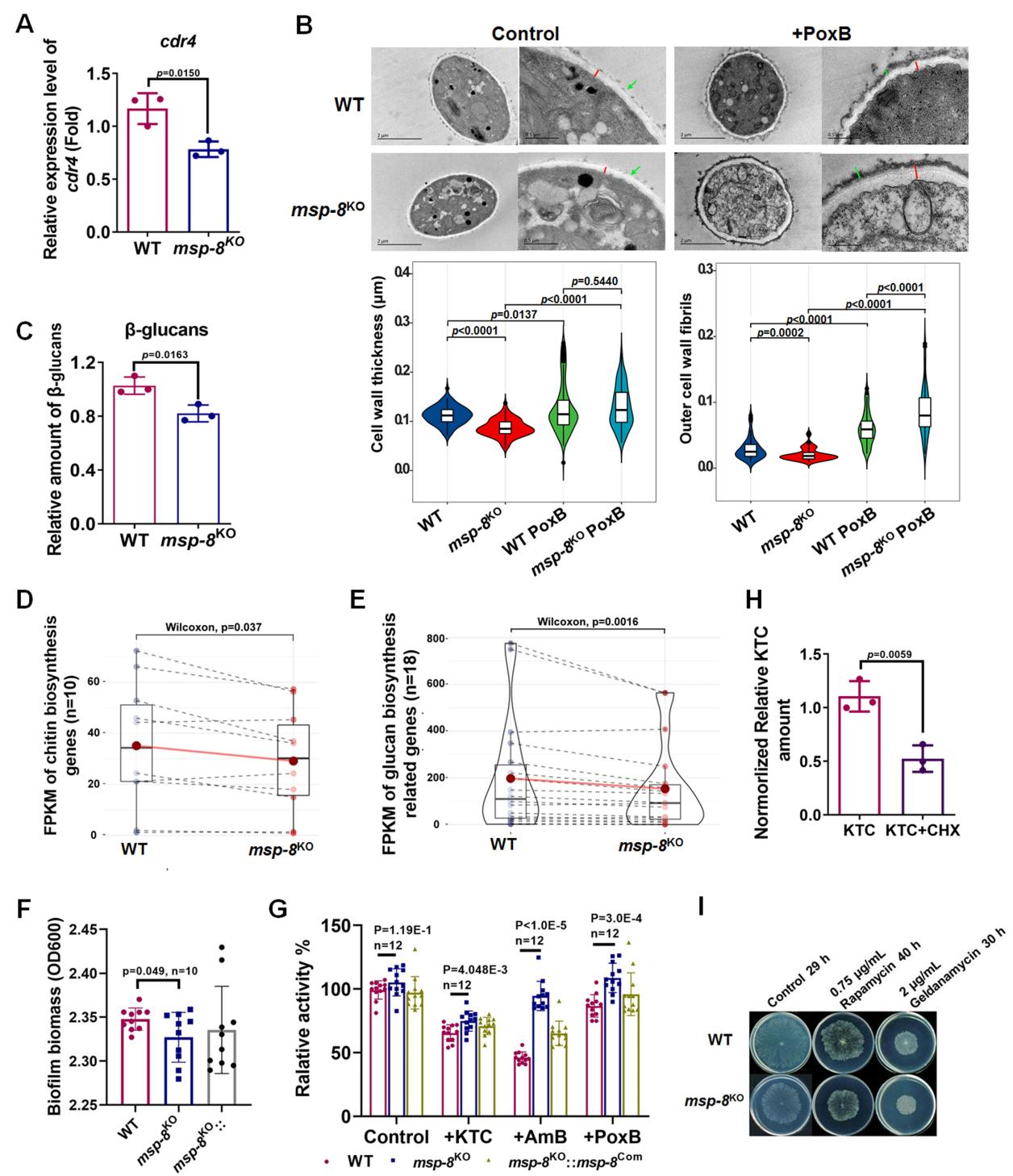
**

**Figure S5.** **(A)** Relative expression levels of the *cdr4* gene in *msp-8*^KO^ versus WT strains, as determined by RNA-seq analysis. The data are presented as mean ± SD with n=3. (**B**) Transmission electron microscopy images of the cell wall and outer fibrils. The violin plot below illustrates the quantitative analysis of the cell wall thickness (indicated by the red line) and outer wall fibril thickness (indicated by the green line or arrow). Box plots show the median and the upper and lower central quartiles. **(C)** Relative amounts of β-glucans in *msp-8*^KO^ and WT strains. The data are presented as mean ± SD with n=3. (**D, E**) FPKM values of chitin biosynthesis genes (**D**), and glucan biosynthesis-related genes (**E**) between *msp-8*^KO^ and WT strains. Each dot indicates the mean of three independent experiments. Statistical significance is determined by the wilcoxon test. (**F**) Biofilm biomass of the WT, *msp-8*^KO^ and *msp-8*^KO^:: *msp-8*^com^ strains. The data are presented as mean ± SD. (**G**) The relative metabolic activity of the biofims in the indicated strains. The data are presented as mean ± SD. (**H**) Accumulation of KTC in *cdr4*^KO^ under KTC stress and KTC treatment with translation inhibitor CHX. The data are presented as mean ± SD with n=3. (**I**) Drug susceptibility test of WT and *msp-8*^KO^ strains to rapamycin and geldanamycin. Statistical significance for (**A**), (**B**), (**C**), (**F**), (**G**) and (**H**) is determined by the two-tailed t-test.

**Table S1. Strains in the study**

| **Species** | **Strains** | **Source** | **Genotype** |
| --- | --- | --- | --- |
| *N. crassa* | *cdr4*^KO^ | FGSC#11238 | hygromycin B resistance (Hph^R^); Oak Ridge (OR) |
|  | 30thC4 | this study | Hph^R^ |
|  | 28thK1 | this study | Hph^R^ |
|  | 29thK3 | this study | Hph^R^ |
|  | 27thK4 | this study | Hph^R^ |
|  | 17thV1 | this study | Hph^R^ |
|  | 26thV3 | this study | Hph^R^ |
|  | 27thV4 | this study | Hph^R^ |
|  | 2k-6 | this study | Hph^R^ |
|  | Wild type (WT) | FGSC#4200 | Prototrophic |
|  | FGSC#2225 | FGSC#2225 | Prototrophic; Mauriceville Texas (MV) |
|  | *msp-8*^KO^ | FGSC#12359 | Hph^R^; *NCU01143* knockout |
|  | *msp-8*^KO^::*msp-8^COM^* | This study | Hph^R^; Chloramphenicol resistance (Chl^R^) |
|  | MSP-8^E749K^ | This study | Chl^R^ |
|  | *msp-8*^KO^*::msp-8*^E749K^ | This study | Hph^R^; Chl^R^ |
|  | *cdr4*^KO^::*msp-8*^E749K^ | This study | Hph^R^; Chl^R^; *cdr4* knockout; *msp-8* point mutation (E749K) |
|  | *cdr4*^KO^::*msp-8*^KO^ | This study | Hph^R^; Chl^R^; *cdr4* knockout; *msp-8* knockout |
|  | 2k-6::*msp-8*^COM^ | This study | Hph^R^; Chl^R^; *cdr4* knockout |
|  | 3×Flag-CYH-2::MSP-8-5×Myc | this study | ChlR; MSP-8-5×Myc overexpression; 3×Flag-CYH-2 overexpression |
|  | 2k-6::*atrf*^KO^ | this study | HphR; Chloramphenicol resistance (Chl^R^) |
|  | *msp-8*^KO^::*msp-8*-GFP^OE^ | this study | Hph^R^; Chl^R^; MSP-8-GFP overexpression |
|  | 2k-6::*atrf-2*^KO^ | this study | Hph^R^; Chl^R^ |
|  | NcRqc1^KO^ | FGSC#19824 | Hph^R^; *NCU03367* knockout |
|  | NcHel2^KO^ | FGSC#11883 | Hph^R^; *NCU02203* knockout |
|  | NcLtn1^KO^ | FGSC#11923 | Hph^R^; *NCU06534* knockout |
|  | *pup-6*^KO^ | FGSC#16283 | Hph^R^; *NCU04195* knockout |
|  | *cem-5*^KO^ | FGSC#16247 | Hph^R^; *NCU01092* knockout |
|  | *cyh-2*^KO^ | FGSC#15677 | Hph^R^; *NCU03806* knockout |
| *F. verticillioides* | Fv7600 | FGSC#7600 | Prototrophic |
|  | Fv*msp-8*^KO^ | This study | Hph^R^; FVEG_08023 knockout |
| *F. fumigatus* | CEA17 | Cheng Jin's Lab | *pyrG^-^* |
|  | Af*msp-8*^KO^ | This study | pyrG^+^; AFUA_2G16140 knockout |
|  | ATCC | Wenxia Fang's Lab | Prototrophic |
|  | Afmsp-8^F367Y^ | This study | pyrG^+^; AFUA_2G16140 point mutation (F367Y) |
|  | 2707 | Wenxia Fang's Lab from the clinic | MSP-8 point mutation (F367Y) |

**Table S2. Primers used for plasmid construction in this study**

| **primer name** | **sequence(5’→3’)** |
| --- | --- |
| For construction of *msp-8*^E749K^ point mutant and its complemented strain | |
| PoMut*msp-8*-5F | GGGCTGCAGGAATTCGATAGAAGAAGAGAGCTCAGCTCCA |
| PoMut*msp-8-*5R | TCAGGCTTCCAACAGCATGTCTTGCTTGGAGATGT |
| PoMut*msp-8*-chlF | CATGCTGTTGGAAGCCTGAATGGCGAATGGAAA |
| PoMut*msp-8*-chlR | GATGTGGTCGGAGGAATAAGGGCGACACGGAAA |
| PoMut*msp-8*-3F | TTATTCCTCCGACCACATCTCTCTCTCTCTTTCTCAGTG |
| PoMut*msp-8*-3R | cggtatcgataagcttgatGCCTGGCTACTCGTAGACTGA |
| For complementation of *msp-8* deletion strain | |
| MSP-8situCom5F | AGCAGAggcgcgccTGAATGATGGACGGGCAGACA |
| MSP-8situComMsp-8R | cggtatcgataagcttgatAGCATGTCTTGCTTGGAGATGT |
| MSP-8situCom3F | GGGCTGCAGGAATTCGATCCTATGACGTTCTGTTCTCCG |
| MSP-8situCom3R | CATTCAggcgcgccT CTGCTGACGGGAAGGAGTTC |
| For construction of MSP-8-MYC fusion-expressed strain | |
| Myc-TrpC-F | gcttcatCGATGAATTCTGACCGGGATCCACTTAACGTTACTG |
| TrpC-Qa-R | CGGGTACCGAGCTCGAATTCGGCGTAGAGGATCCTCTAGAAAGA |
| msp-8-tag-F | GGTCTTGATGCTCATCGATTTAAAGCaATGGAGC |
| TrpC-Pcb-R | cggtatcgataagcttgatGGCGTAGAGGATCCTCTAGAAAGA |
| pBM-cfpF | taagcttgatatcgaattGTTGCGTTTCTGACTATCCG |
| cfp-msp-8R | TTGGCCATAAGTGTCAAAGACTGGAGGTG |
| cfp-msp-8F | TTTGACACTTATGGCCAAAAAGGGAACCAAG |
| msp-8-tagR | AATCGATGAGCATCAAGACCATCAAGC |
| For construction of *Flag-CYH-2* fusion-expressed strain | |
| 1532pCcg1FlagF | CCGGGCTGCAGGAATTCGATTTCGTTCAAAGCCACATCAC |
| 1532Ccg1FlagR | ACGGTATCGATAAGCTTGATGACTCACTATAGGGCGAATT |
| Flag-3806F | GAGGCGGCGGAGGCGGCGCGATGgtatggcaattgctgca |
| Flag-3806R | ACTAGTTAATTAATGGCGCGcgagagatgagatctgttggg |
| Flag-0315F | GAGGCGGCGGAGGCGGCGCGATGGCCAgtatgttgtgc |
| Flag-0315R | ACTAGTTAATTAATGGCGCGaaagagtatcaaaaccttcttgt |
| For construction of *MSP-8-GFP* fusion expression strain | |
| Pcb-cfpF | GGGCTGCAGGAATTCGATGTTGCGTTTCTGACTATCCG |
| Cfp-msp-8R | tccgcctccgcctccgcctccAGCATCAAGACCATCAAGC |
| msp-8-gfpF | ggcggaggcggaggcggaatggtgagcaagggcgaggaG |
| gfp-TrpCR | GATCCCGGcttgtacagctcgtccatgccg |
| gfp-TrpCF | ctgtacaagCCGGGATCCACTTAACGTTACTG |
| TrpC-PcbR | cggtatcgataagcttgatGGCGTAGAGGATCCTCTAGAAAGA |
| For construction of *atrf* deletion strain | |
| *atrf*^KO^ 5F | GGGCTGCAGGAATTCGATGCTTACAAATTCGAGCCTCC |
| *atrf*^KO^ 5R | TCAGGCTTCCAACGACTGAGTTGTGTCAAGACG |
| *atrf*^KO^Chl-F | CAGTCGTTGGAAGCCTGAATGGCGAATGGAAA |
| *atrf*^KO^Chl-R | CCTACGTCGGAGGAATAAGGGCGACACGGAAA |
| *atrf*^KO^ 3F | TTATTCCTCCGACGTAGGCATGTCGTACCTTGG |
| *atrf*^KO^ 3R | cggtatcgataagcttgatGTCCCTTGACCTTGTCATCC |
| For construction of *atrf-2* deletion strain | |
| *atrf-2*^KO^ 5F | GGGCTGCAGGAATTCGAT ATCCTACGCTTCGGTAGACG |
| *atrf-2*^KO^ 5R | TCAGGCTTCCAACGCTATATGCCACACTCTTCG |
| *atrf-2*^KO^Chl-F | ATAGCGTTGGAAGCCTGAATGGCGAATGGAAA |
| *atrf-2*^KO^Chl-R | CGAGGGTCGGAGGAATAAGGGCGACACGGAAA |
| *atrf-2*^KO^ 3F | TTATTCCTCCGACCCTCGTACGAACTACCTACC |
| *atrf-2*^KO^ 3R | cggtatcgataagcttgat CATCTGTACCGTTACCTTCG |
| For construction of *msp-8* deletion strain (*A. fumigatus*) | |
| Af*msp-8*^KO^-5F | gataagcttgatatcgaattCTGAGAATGTAATCGGTCAAGAGG |
| Af*msp-8*^KO^*-*5R | AGTCTAGAGCGTGGAGCAACTCGGGATAT |
| *msp-8-pyrG*-F | TGCTCCACGCTCTAGACTTCCTAATACCGCCT |
| *msp-8-pyrG*-R | CCTGTCGCCCCGGATCTCTAGAGGTAAGTAATC |
| Af*msp-8^KO^*-3F | GAGATCCGGGGCGACAGGGTGATTGAGAT |
| Af*msp-8*^KO^-3R | ccccgggctgcaggaattGCCAGGCTGATGCTGAGAAA |
| For construction of *erg5* deletion strain (*F. verticillioides*) | |
| Fv*msp-8*^KO^-5F | GGGCTGCAGGAATTCGATATGTAAAAGGCTCGCTGGAT |
| Fv*msp-8*^KO^*-*5R | CATCTTCTGTCGGTGGTTTGTTGGTCGTGTTG |
| *msp-8-Hph*-F | CAAACCACCGACAGAAGATGATATTGAAGGAGC |
| *msp-8-Hph*-R | AACAGATACGGTCGGCATCTACTCTATTCCT |
| Fv*msp-8^KO^*-3F | TGCCGACCGTATCTGTTCATGCCTGTACTTCGG |
| Fv*msp-8*^KO^-3R | cggtatcgataagcttgatGGTTGATTTGCTTTAGGGTGCT |

**Table S3. Gene-specific primers used for qPCR**

| **Gene** | **Locus No.** | **Forward primer(5’→ 3’)** | **Reverse primer(5’→ 3’)** | **Ref** |
| --- | --- | --- | --- | --- |
| *β-tubulin* | NCU04540 | CCCAAGAACATGATGGCTGCTTCT | TTGTTCTGAACGTTGCGCATCTGG | **Zhou et al. 2022** [S1] |
| *erg11* | NCU02624 | AAATCGATTACGGCTACGGTCTCG | TATCGCTACCATCCACGTTCCTGA |  |
| *cdr4* | NCU05591 | GCTTTGGAAATGGATGGTGACGCT | AAATGCAGAGGGCGGTCTTAGAGT |  |
| *atrf* | NCU08056 | GGCGCTCGTTTACTTCTT | TCTTCCTTCTCCTCCTTCTT | **This study** |
| *atrf-2* | NCU10009 | GGAGTACATGGAACCTTTCTTC | CAAATCCCTCCACCTGTTATC |  |
| *msp-8* | NCU01143 | ACCGTTGATGGGAAGAATG | CAGCGTTGTCGTCATAGTAG |  |

**Table S4. Primers and enzymes used for CAPS assay** [S2]

| **Contig-Position** | **Forward primer(5’→ 3’)** | **Reverse primer(5’→ 3’)** | **Enzyme** |
| --- | --- | --- | --- |
| 1-304795 | GAGGGCATAGATGCACAGGT | TGTTTGGCTTTTCGGACTTT | AluI |
| 1-678860 | ATGACTATGATGCGGCTGGT | GTTGAACGTTCTCGCCATCT | BstUI |
| 1-1234469 | AGGGCCCAGGTAGCGTAG | CCGTGCAAGGACAGAGAGA | MseI |
| 2-76236 | GCGATGCAACTGTGTCATGT | GCTTAAGCGACAGGGTAGGT | MspI |
| 2-479688 | AACCCGGATGAGGAGACAG | TCAGTCACGTCAACGAGAACA | AluI |
| 2-850994 | GCGTTAGGGTTCAAGCTACG | TTTTTCGCTTCGATTTTCTACC | ApoI |
| 2-1341552 | TTGTGGCGATAAATGGGATT | GATCTAAACCCCCTCGAACA | MseI |
| 2-1676969 | GAGAGTTTTGTCGCGATTGA | GCCTTCTTCCTTCCATACCC | Tsp509I |
| 3-230972 | GACACTTCGAAAACGGGAAC | AAAAGCGAATAATCAAAGAAAACG | AluI |
| 3-753217 | CCAAGTTGGGCTTTGCTG | GATTAACCCGCTATGCAACG | MboI |
| 3-1219214 | GCCAGAAGAACTTGGTTGGA | GCTTGGGTTGATGGGTGATA | BstUI |
| 4-190784 | ACATATCATTCCCGCCACTC | TCTTTTGCTTGACGTTGTCG | MseI |
| 4-334884 | GACTGTAGAGACGTTGGGTAGG | TGAAAGTGAGAAAGTCTGGAGGT | NlaIII |
| 4-787747 | CTACCAGGCCTACTGGGACA | GCACTCACGGTAAGCGAAA | BstUI |
| 5-62411 | CTGAGGCACCACAACAACC | TTGCGTTACAAAAAGGTGGA | Tsp509I |
| 5-501130 | AACCCGGTCATCGTGCTC | CCTGGGCATAGGGCGTAG | MseI |
| 5-950333 | AACAAGCAGAAGGGCATCAC | TGTAAGGGGGAATGGGGTAT | HaeIII |
| 6-118243 | CTCGAGGATTGGATGTAGCC | GCATAGATTGCCAGCCAAAA | MseI |
| 7-311101 | ACAAAGTCACTTGAAACAGAAAAA | CTTGTGGATGGAAGGGGAAC | Tsp509I |
| 7-815075 | CCAAGTCACAAAGTCGTCCA | GCACTCCACTCCCTCGTACT | BfaI |
| 8-65825 | TCTCGTCGCCCTACCACTAC | GGGGTCGATGTTGATGATG | MseI |
| 8-273655 | GTGGTCGCATGAGTTCTTGA | CACCCTTTCCACCAGCAC | Tsp509I |
| 8-573568 | GGTACCTCCGTGAAGACTCG | CGTTCATAAACAATGTAGTGCTAAG | MseI |
| 8-877559 | AAAGGCCGGTACCCGTTA | GCTTCTCGTGGATGCAGTTC | HhaI |
| 9-218368 | CCGTAGCGAGTCCCAGTAA | GACCCAGACACTTGCCATTT | Tsp509I |
| 9-550012 | GATGAGGAGACGGACGACAT | TACCCCGACTGACAAATTCC | NlaIII |
| 9-590198 | GGAGGGTTTTCGTCTCGTTT | TGCACAGACAATATGTGTTCAAT | MboI |
| 9-619665 | TTCAGTACTGGTCCGAAGTGG | ATGTTGGCCAAAAGGAACTG | BstUI |
| 10-23305 | AGATTACCTGCTGCGCTGTG | AGTGCACCATGGCGTCTG | RsaI |
| 10-303154 | GCTCAGATCGGTGCCAAG | CGTCCTGTAACACAATGCTCA | MseI |
| 10-866822 | TTTCTCCGGCCGACATAC | CGAGGGCGTAGAAGGATATG | MseI |
| 11-131404 | CCATGCTGACCCTTTTCTCT | TCGACCTTGGTGAGATCCTT | RsaI |
| 11-467346 | CCTGTGAGGATCAGAGGATCT | CGTGCTGATTGGTCACGTC | TaqI |
| 11-631805 | GCTTGGTAACGCTAGACAGGTT | TAGTGGCAGTACCGGCTTG | MseI |
| 12-126871 | GGCAGCGACAACGAGAATA | ATGAAGTGTCGCAGTGTCTG | MseI |
| 12-375933 | AAGGTCGGGTTGTTAGACGA | GGCTGAGGGTGGATAGCTAGA | TaqI |
| 12-735541 | CATCCAGGAGCTCTTCAAGC | AGGGGTCAACAACTAGAGTGTCT | Tsp509I |
| 13-77852 | CTTCTTCTAGCGGCGGAAGT | TCAAAGTTGCCGTGGTTAAA | BstUI |
| 13-240510 | TTCACCATTGAGCTGTTTGC | ATCTATTCTTGTCAAAGATTTCCAC | MseI |
| 13-572669 | GGATTCCTCCTGACCTTGC | TGAACTTCTCAATAGGCGTAAAG | MboI |
| 15-332745 | CCGGTGATTATTTCTATGTGG | GAGAATCGTTTCCTCGCTTG | Tsp509I |
| 15-519997 | CCCGTTAGGTACCTCTGCAC | GGCGCTTACTTTGGTATTGC | MboI |
| 16-254464 | AAGACGTCGGATTCCCTTG | CGACGAGGATCCATTCTTCT | AluI |
| 17-74838 | GCGAAGGTCATGTTCGACTT | CCGCCCATAGATGTTGAGTC | AccI |
| 17-604962 | TCCACGGAGACCGTTCAG | ACTTGGAGTGCGGATAGCTC | ApoI |
| 18-34228 | AACAGATGGCTTCACAACAGG | TGGCTTCTCATCGAGAGTCTG | MspI |
| 18-217295 | TCAGAAGGCTTCCTCGTTTG | GTTCAAGCTGGTCGCAGAAG | NlaIII |
| 19-628633 | TGGACCCTGTACTTCATGTGG | TGTGTGTAGGCTGCAACTGG | MseI |
| 20-69612 | ACTCAACCGCATCCTCGAC | ATTTCCCTCGCAAACAAATC | Tsp509I |
| 20-652343 | CCAAGGATGACCTGAAGTGG | AGAGCGTGGACGAACATGG | MseI |
| 21-61899 | CTTTGGTTCTTGGCGAACAC | CGCGGAAGAAAGGAAAACTT | MseI |
| 21-189922 | AATGGGTGATGGACCAGTCT | CTGAACGCCCAGTCCTCTAA | NlaIII |
| 21-316319 | CTTGTCCAAGCCTCTTCTGG | CACCAAAAGGACCATCGTG | MseI |
| 22-344646 | AGTTCGTCAAGAGCGTCGAG | TCCGTTGTCCATTCATTGTG | Tsp509I |
| 23-20606 | GATCCCATCAACATGTCCAA | AGCGAGAAGAGGAGGGTGAT | Tsp509I |
| 23-385933 | CCCGACGTGAAGTCAAAATC | TGAGTTGGGACGTACTGATGG | HaeIII |
| 24-158443 | AGACAAGGAATGCGGTTTGT | CGGTGGGATCTTCTCGTATC | TaqI |
| 24-188580 | ACGGTGATCCCAACCACTT | TGCTCATCAAAACAAAGGTCA | MseI |
| 24-488563 | GCGAAATGCCTCCTAATTCC | GGCCAAAATCCATCATCATC | NlaIII |
| 25-126882 | AACGTCAACCACCCGTTAAG | CGCTGTCCGTAGAGGTTCTT | ApoI |
| 27-86887 | TACACCAACCAACCATGCAG | GCAGCTTGTAACGCTTGATG | Tsp509I |
| 29-231038 | GTACGTTGCACCCATCACAC | GAGCCGCCTTTCGTAATAGA | Tsp509I |
| 30-179811 | CAATACAGGGACCACCTTGTG | AGTGGACGTGGTCGCTTAAT | Eco0109I |
| 30-330635 | CTCTCGTAAGGGACCCCAAG | CGTCAACAAAGGGGTAAAGC | NlaIII |
| 32-23669 | CACCATGAGAGGTGACACGA | CGGTAGTTTTGCGTTGCATA | TaqI |
| 32-148324 | GGGGTTTGATTATGGGTGAG | CCTTCGTGGTCGGTCTGAT | MseI |
| 32-312562 | GTCTGTGTCCTGTTCCACCA | AACTGAAAGCCGCTTGCTC | AluI |
| 33-153414 | TACTGCTCTGTTCGCTCCTG | TGATCCGAATAGGAAAGTGGA | NlaIII |
| 34-229394 | TACCGCCGATTCTATCAAGG | TACGGTTTGAGAGGGAGGTG | MboI |
| 35-256795 | GAAGTTCCAAGCCCTGAACA | CCGACCAAATCGTAGAGCAC | RsaI |
| 36-250078 | CTTCACTGAGGGTGCCATCT | CTGTTGAGAATGAGGCAGGA | MseI |
| 37-122184 | CCATTCAGGGAGGTAGACCA | TAAATGAAGCCCCATGTCCT | MseI |
| 39-34936 | CTATCCCATTCCCCCAACTT | CCCTTTGGCTTTCCAAAAAT | Tsp509I |
| 40-149468 | CAGGTGAACAACTCGACCAC | ATCCTTCCTCTCCTCCCACA | MseI |
| 41-87330 | CTGCCTTCCACTTTGGTCAT | GTCGGGTAAGGATCATCCAC | TaqI |
| 42-79510 | GTTCAGCCCAGCTTCCCTAT | GTGAGCCAGATGACGTTGTG | Tsp509I |
| 42-203039 | GTAAGATCGGAGCCCATTCA | AGGAGTGGTTTTCACGTTGG | HaeIII |
| 46-192380 | ACTTGAGCAAGAAGGCGATG | ATCCGTAATCTGCCCAATCC | AluI |
| 46-275448 | CGACCCTCTTGCAGTTTCTC | GTATCGGGCAGCGCATCT | MseI |
| 48-38247 | TTCGACTACGCCATTGTTTTC | CCTCCTTGGCTTGGTTCTG | RsaI |
| 49-100367 | AGTCCGAGGCTGTCACTGAT | CACCGGAAAGAGCAGAGATG | MseI |
| 53-154343 | CGTTGAGATCGTTGCGTGTA | ACGTCAACAAGATCCCGAAG | MseI |
| 53-225365 | GTGGGAGCCGTTGGAAAG | ACGCTAGCATCTTGCTGTTG | Tsp509I |
| 54-138140 | CCGTTTGGCTAGAAAATTCG | GAGGTCTCGAGGAAGGGAAT | MseI |
| 64-106352 | CTTCCCCGTGACCCTCTG | TCCGTCATATATGTACAGCCATA | TaqI |
| 67-33228 | ATTCCTGCCCAGAAGGACTC | GCTGAAGCACATGGAAAGGT | MseI |
| 71-61194 | AGCACAACACACCAGATTCG | CGGTAAGTGGGGAGGTAAGC | Tsp509I |
| 72-66244 | AGGACAACACCTTCACTCACC | CAAGCAAAACCCATCCATTT | MseI |
| 79-21889 | CATTCTTCCCCGCTGCTA | ACCCAGGATGCAGAACAGAC | NlaIII |
| 79-61346 | CTCATGAAGCGCTCTACCG | GCCGCCAATGTATACAGAAGA | MspI |
| 81-93277 | CGCTATGCGTCAACACAAAA | GTCTGCCAAAGCGACACTG | NlaIII |
| 85-42557 | ACTCCGTCGTCAACGATACC | TCATGTGAAAGAAGGATGTCG | HaeIII |

Supplemental References

[S1] M. Zhou, C. Hu, Y. Yin, J. Wang, S. Ye, Y. Yu, X. Sun, S. Li, *J. Fungi* **2022**, *8*, 198-217.

[S2] R. Lambreghts, M. Shi, W. J. Belden, D. DeCaprio, D. Park, M. R. Henn, J. E. Galagan, M. Baştürkmen, B. W. Birren, M. S. Sachs, *Genetics* **2009**, *181*, 767-781.
