# Supplementary material for: Suppressed Protein Translation Caused by MSP‐8 Deficiency Determines Fungal Multidrug Resistance with Fitness Cost
Source: Adv Sci (Weinh). 2024 Dec 16;12(6):2412514. doi: 10.1002/advs.202412514 (PMC11809369; doi:10.1002/advs.202412514)
Supplement: Supplementary file 2 — Supporting Information [file ADVS-12-2412514-s002.zip › advs202412514-sup-0002-Data/File S2.docx]

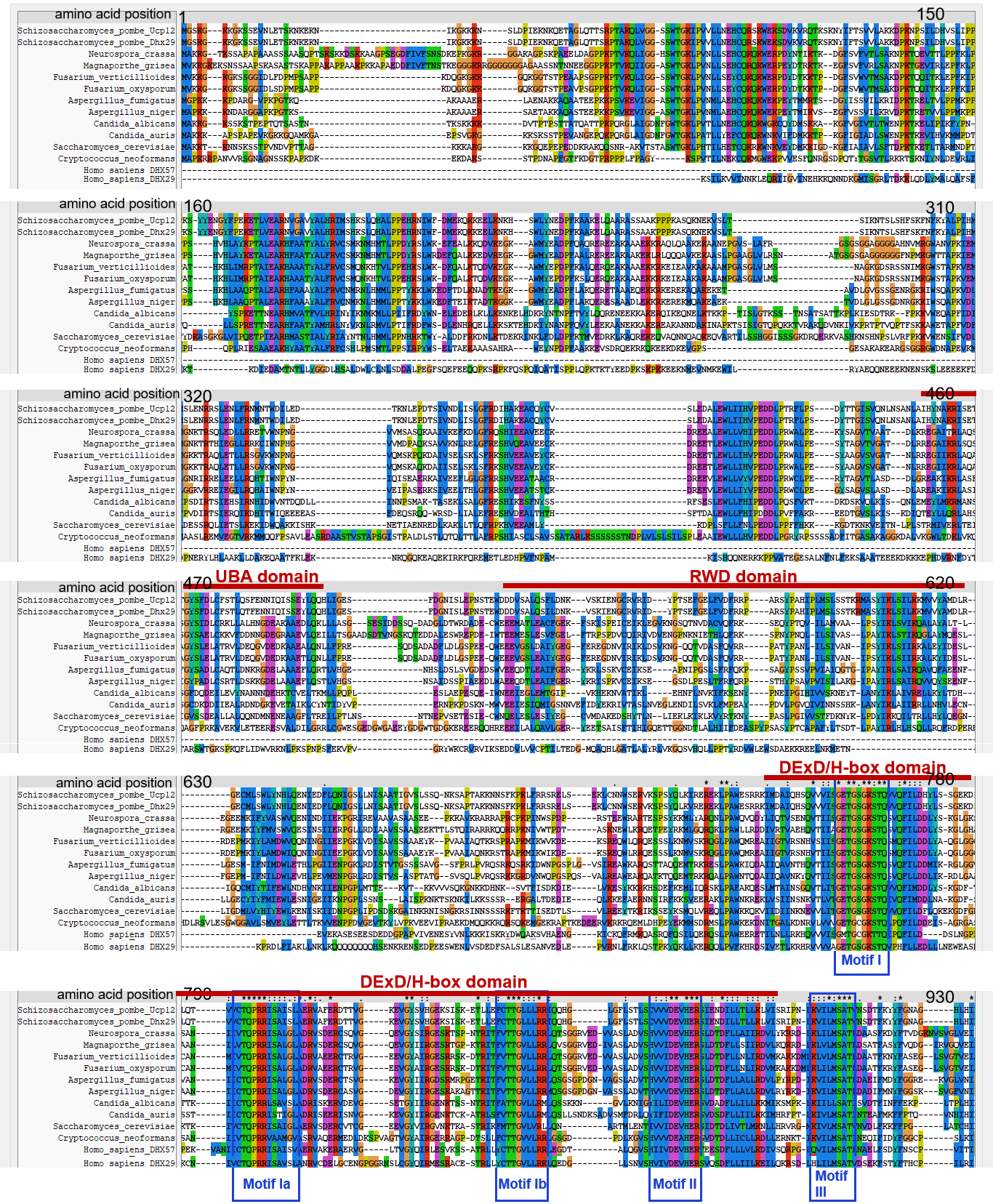


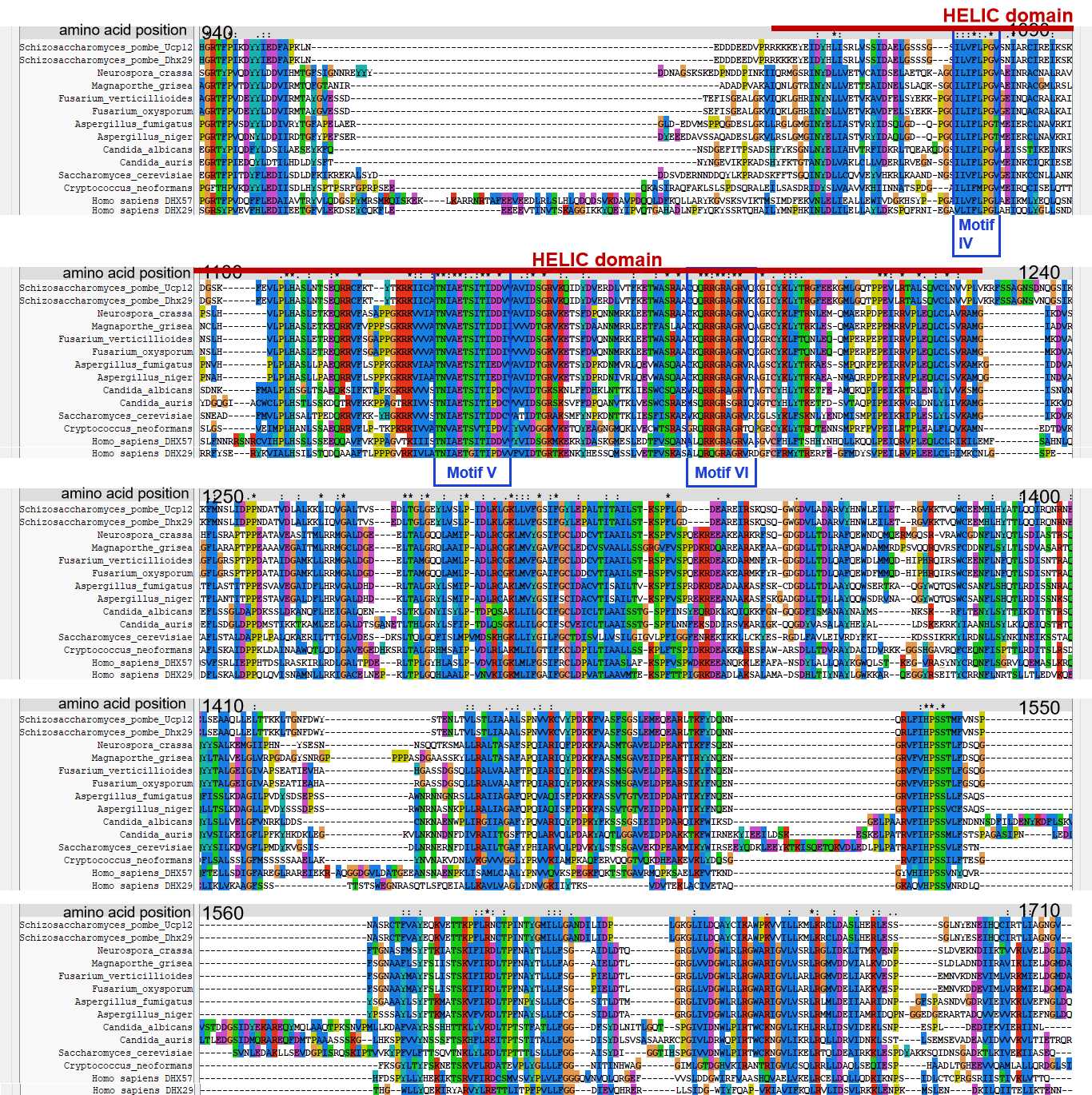


**File S2**. Alignment of MSP-8 homologs and analysis of conserved amino acid residues in *Neurospora crassa (EAA31814.2)*, *Magnaporthe_grisea* (XP_030985144.1), *Fusarium verticillioides* (RBQ89864.1), *Fusarium oxysporum* (RKK76910.1), *Aspergillus fumigatus (*XP_755949.1*)*, *Aspergillus niger* (XP_025454843.1), *Candida albicans* (XP_719912.1), *Saccharomyces cerevisiae* (NP_013523.3), *Schizosaccharomyces pombe* (KAL2312805 and NP_588050.1), *Candida auris* (XP_028890719.1), *Cryptococcus neoformans* (XP_024512306.1), *Homo_sapiens* (AAH65278.1 and CAH56172.1).
